# Development and validation of a machine learning-based model for predicting amputation risk in patients with lower extremity arterial disease and diabetes

**DOI:** 10.3389/fcvm.2026.1893875

**Published:** 2026-10-20

**Authors:** Lu Tang, Liang Wang, Jing Li, Xiaoyu Liu, Qin Chen, Pan Song, Xiaoyan Quan, Qingran Li, Gengliang Liu, Yang Liu, Chi Cui, Meihong Shi

**Affiliations:** 1 School of Nursing, Southwest Medical University, Luzhou, Sichuan Province, China; 2 The Chengdu First People’s Hospital, Chengdu, Sichuan Province, China; 3 Health Management Center, The Affiliated Hospital of Southwest Medical University, Luzhou, Sichuan Province, China; 4 Department of Spine Surgery, The Affiliated Hospital of Southwest Medical University, Luzhou, Sichuan, China; 5 Department of Critical Care Medicine, Dazhou Central Hospital in Sichuan, Dazhou City, Sichuan Province, China; 6 Hospital of Chengdu University of Traditional Chinese Medicine, Chengdu, Sichuan Province, China; 7 The Third People’s Hospital of Chengdu, Affiliated Hospital of Southwest Jiaotong University & The Second Affiliated Hospital of Chengdu, Chongqing Medical University, Chengdu, Sichuan Province, China

**Keywords:** lower extremity arterial disease, diabetes, amputation, machine learning, clinical prediction model

## Abstract

**Background:**

Patients with lower extremity arterial disease (LEAD) complicated by diabetes face substantial amputation risk. Pre-amputation death acts as a critical competing event that distorts risk estimation. Validated personalized prediction tools for this population remain scarce and hinder precise clinical interventions. This study aimed to develop and validate two complementary interpretable models for 12-month amputation risk in patients with LEAD and diabetes to support clinical decision-making.

**Methods:**

This retrospective multicenter study enrolled 1,919 patients with LEAD and diabetes from four Chinese hospitals (January 2018-September 2024), with follow-up ending in September 2025. The primary outcome was 12-month amputation (major or minor). Prior to model construction, candidate predictors were screened via LASSO regression, random forest classifier and Boruta algorithm. Ten machine learning algorithms were then trained and evaluated by discrimination and calibration metrics. SHapley Additive Explanations (SHAP) was applied for interpretability. A Fine-Gray competing risk regression model was built using the same screened predictor set.

**Results:**

Patients were split into training (*n* = 967), internal validation (*n* = 414), and external validation (*n* = 538) cohorts. The 12-month amputation rate was 14.0%. Median follow-up was 12 months; mean age was 72.7 ± 9.5 years and 65.7% were male. The optimal GraBoost model achieved internal validation AUC = 0.860 and external validation AUC = 0.828, incorporating 14 clinical predictors, including: Rutherford classification, financial burden, age, Barthel Index, diabetic foot, statin, fibrinogen (FIB), neutrophil (NEUT), residence, prealbumin (PALB), C-reactive protein (CRP), activated partial thromboplastin time (APTT), body mass index (BMI) classification and wound. SHAP revealed higher grade Rutherford classification, lower Barthel Index, younger age, wounds, diabetic foot, and elevated FIB, APTT, CRP, NEUT correlated with higher amputation risk. Urban residence, normal BMI classification, lower financial burden, statin use and higher PALB were protective. Shared predictors showed consistent directional effects across GraBoost and Fine-Gray models. A dual-model web-based calculator was constructed for risk assessment.

**Conclusion:**

We developed and validated complementary interpretable GraBoost machine learning and Fine-Gray competing risk models for 12-month amputation risk. The dual-model web calculator may help clinicians identify high-risk patients and guide clinical management.

## 1 Introduction

Lower extremity arterial disease (LEAD) is a chronic vascular disease. It is caused by atherosclerosis and features stenosis or occlusion of the lower extremity arteries (1). Globally, approximately 236 million people are affected by LEAD, with a prevalence rate reaching 10.0% among individuals aged 60 and above in China (2). Meanwhile, the prevalence of diabetes in China has risen markedly in recent years (3), with an estimated 113.9 million adult diabetic patients, accounting for 24% of the global cases (4). Diabetes is a major risk factor for the development and progression of LEAD (1). Chronic hyperglycemia induces vascular endothelial cell injury and inflammatory responses. As a result, it accelerates intimal plaque formation, which in turn promotes arterial wall sclerosis, stenosis, or occlusion (5). Atherosclerotic progression exacerbates lower extremity circulatory impairments in diabetic patients, creating a reciprocal pathogenic cycle. Consequently, diabetic patients exhibit fourfold higher LEAD incidence than non-diabetics (6–8), with amputation rates of 13%–48% (9, 10) and mortality rates reaching 23%–52% (11) in comorbid cases. Given the high risk of amputation and mortality faced by patients with LEAD and diabetes, early and accurate risk prediction is essential to enable precise management. Of note, among this patient population, mortality may precede amputation, creating a competing-risk situation that complicates accurate estimation of amputation risk (12).

Machine learning (ML) has emerged as a powerful tool for data mining and analysis, and as an increasingly important technology applied to clinical prediction modeling (13). Research has shown that ML can outperform traditional statistical methods and risk scores in certain scenarios, particularly in handling multi-dimensional (14, 15), complex datasets and characterizing intricate relationships between predictors and outcomes (16, 17). Currently, studies on ML predictive models predominantly focus on single-disease populations, such as the risk of diabetic foot amputation (18–22) or amputation risk in LEAD (23–26). However, for the comorbid patients with LEAD and diabetes, no validated amputation risk prediction model exists to date. Existing research has been primarily limited to identifying risk factors for amputation in this population (27–29), and a comprehensive predictive tool has yet to be established. Furthermore, existing amputation-risk prediction studies mostly adopt binary classification ML frameworks (21, 30, 31).

Binary classification ML models can produce intuitive risk outputs and support fine-grained individual level interpretability via SHAP. However, binary frameworks do not explicitly account for the competing event of pre-amputation death. Competing risk survival analysis appropriately handles competing events, yet it offers limited individual level interpretability and generally relies on nomogram-based scoring. Given the respective strengths and limitations of the two approaches, along with the more complex clinical profiles exhibited by comorbid patients, integrating both models into a unified tool facilitates more comprehensive clinical risk assessment and assists clinicians in risk evaluation and the formulation of preventive strategies.

## 2 Methods

### 2.1 Study design

Multicenter retrospective cohort study.

### 2.2 Patient cohort

The training cohort included patients with LEAD and diabetes. These patients were retrospectively recruited from the vascular surgery departments of three Chengdu hospitals (Hospital of Chengdu University of Traditional Chinese Medicine, Chengdu Integrated Traditional Chinese and Western Medicine Hospital, The Third People's Hospital of Chengdu) between January 2018 and September 2024. The dataset was partitioned into 70% training and 30% internal validation subsets. A geographically distinct external validation cohort comprising patients from Southwest Medical University Affiliated Hospital's vascular surgery department was established for model robustness assessment.

Inclusion criteria: (1) LEAD confirmation following diagnostic guidelines (1): 1) Age >40 years; 2) Presence of high-risk factors such as smoking, diabetes, hypertension, or hyperlipidemia; 3) Clinical manifestations of LEAD; 4) Weakened or absent arterial pulsation in the distal ischemic limb; 5) Ankle-brachial index (ABI) ≤ 0.9; 6) Imaging studies revealing stenosis or occlusion of the corresponding arteries (A clinical diagnosis of LEAD can be made if the first four criteria listed above are met). (2) The patients had been previously diagnosed with diabetes (32);

Exclusion criteria: (1) Incomplete clinical documentation; (2) Concurrent diagnosis of malignancy; (3) Severe cognitive impairment or psychiatric disorders; (4) Inability to provide signed or oral informed consent, inability to be contacted during follow-up, or inability to accurately report outcomes or provide reliable medical information.

### 2.3 Ethical considerations

All participating centers granted ethical approval: (1) Ethics Committee of Hospital of Chengdu University of Traditional Chinese Medicine (Approval No. 2022KL-091); (2) Ethics Committee of Chengdu Integrated Traditional Chinese and Western Medicine Hospital (Approval No. YNYJ-2024-019); (3) Ethics Committee of The Third People's Hospital of Chengdu (Approval No. 2024-S-185); (4) Clinical Trial Ethics Committee of The Affiliated Hospital of Southwest Medical University (Approval Numbers: KY2024241).

### 2.4 Data collection

First, a systematic literature review was conducted using databases such as PubMed, Web of Science, and CNKI, which identified 75 potential risk factors for amputation in patients with LEAD and diabetes (Supplementary Table S1). Second, a panel discussion was held to exclude factors that were clinically inaccessible. Subsequently, data were extracted from the hospital's electronic medical records. Patients with >25% case-level missing data were excluded. Multiple imputation using the fully conditional specification (FCS) method was applied to variables with ≤25% missing data on the entire pooled dataset prior to model construction, and categorical variables were encoded. Variables with >25% missing values were removed to generate the final set of variables for analysis. The resulting variables and their descriptions are provided in Supplementary Table S2.

### 2.5 Outcome

The primary study endpoint was 12-month amputation after enrolment, defined as either major amputation (above-knee or below-knee amputation) or minor amputation (toe-level amputation). Within the 12-month follow-up window, three types of observations were defined: (1) Index event: occurrence of amputation; (2) Competing event: death occurring before amputation without limb-loss; (3) Censoring: patients alive without amputation at 12-months, or loss-to-follow-up before the 12-month time-point. Outcome adjudication criteria: (1) Amputation diagnosed by two surgeons during hospitalization or outpatient follow-up; (2) Amputation self-reported by the patient and subsequently verified by two surgeons through review of submitted medical records.

### 2.6 Sample size

The sample size was calculated using the method proposed by Riley et al. (33). We adopted the lower boundary (13%) of the reported amputation incidence (13%–48%) among patients with LEAD and diabetes. Furthermore, the minimum expected: area under the receiver operating characteristic curve (AUC) value and the candidate predictor parameter for this study were set at 0.8 and 15, respectively. Substituting the above parameters in to the code yields a required number of amputation events for model development of 120, and a minimum sample size of 918 patients.

### 2.7 Prediction model

#### 2.7.1 Feature selection

Feature selection was performed exclusively in the training cohort to avoid information leakage. Three complementary algorithms, including the least absolute shrinkage and selection operator (LASSO) logistic regression, random forest classifier, and Boruta algorithm, were applied to screen candidate predictors. Different from single-threshold screening strategies, this multi-algorithm framework improved the robustness and stability of feature filtering.

1. LASSO logistic regression is superior to conventional methods, owing to its use of an L1-norm penalty for regularization and feature selection. This function automatically selects features by shrinking unimportant coefficients to zero, thus selecting a parsimonious subset of variables. The regularization strength was determined by the *λ* parameter, which set the penalty magnitude in the likelihood function. Optimal *λ* was identified through five-fold internal cross-validation with stratified random partitioning. The one-standard-error rule was applied to select parsimonious models within minimal deviance range. LASSO implementation utilized glmnet package in R environment.
2. The random forest classifier, implemented in Python 3.0, was employed to train the model and extract feature importance. The random forest algorithm calculates importance scores for each feature and ranks variables accordingly.
3. Boruta's algorithm is a method used to determine the most important features in a dataset. The Boruta algorithm was used to identify the most important features by comparing the Z-value of each feature against that of “shadow features”. By duplicating all real features and shuffling them sequentially, the Z-value of each attribute is obtained from a random forest model in each iteration, and the Z-value of shadow is created by random shuffling of the real features. A real feature is regarded as “important” if its Z-value is greater than the maximal Z-value of shadow features in multiple independent trials (34, 35).

Variables identified by at least two of the three methods were retained as the final predictive features. Notably, the identical set of screened predictors was used for subsequent binary machine learning modeling and competing-risk modeling, ensuring that differences in model performance originated from distinct statistical frameworks rather than inconsistent variable inputs.

#### 2.7.2 Development and evaluation of the model

1. Ten ML algorithms, including random forest (RF), logistic regression (LR), decision tree (DT), naive bayes (NB), artificial neural network (ANN), K-Nearest neighbors (KNN), eXtreme gradient boosting (XGBoost), gradient boosting (GraBoost), gradient boosting with categorical features support (CatBoost) and light gradient boosting machine (LGBM), were implemented via Python 3.0/Jupyter Notebook with hyperparameter tuning. A two-dimensional evaluation framework assessed discriminative capacity AUC, accuracy, precision, recall, F1-score, specificity, log-loss, matthews correlation coefficient (MCC) and calibration performance (brier score, calibration curves). Importantly, AUC values above 0.80 are generally considered clinically useful, whereas values below 0.80 are considered to have limited clinical utility (36). Meanwhile, we applied DeLong's test to compare the AUC values among different machine learning models. The Brier score ranges from 0 to 1, with values closer to 0 indicating smaller error and better overall performance. A Brier score ≤ 0.1 indicates excellent predictive performance, while values between 0.1 and 0.2 are regarded as clinically acceptable (37). Receiver Operating Characteristic curve (ROC) and calibration curves were generated across validation sets, enabling visual comparison of model performance metrics. Although multiple metrics were reported descriptively, the optimal model was selected primarily based on calibration adequacy and maximal AUC to ensure robustness and generalizability.
2. To address the inherent statistical limitation of conventional binary classification models, a competing-risk model was constructed using exactly the same set of screened predictors as the binary machine-learning models. In this study, the study endpoint was strictly restricted to 12-month amputation after enrollment. In the competing risk framework, lower extremity amputation was defined as the index event, while all-cause death occurring prior to amputation was defined as the competing event. Patients who survived without amputation at the 12-month time point and those lost to follow-up before the endpoint were regarded as censored cases. To evaluate model performance, we calculated the 12-month time-dependent AUC and Brier score and generated calibration curves to assess discrimination and calibration. In addition, Gray's test was used to compare the 12-month cumulative incidence of amputation across the three cohorts.

Different from binary machine-learning models, which uniformly code pre-amputation death as non-amputation events and may lead to biased risk estimation, the competing risk method appropriately accounts for mutually exclusive competing events. This approach accurately estimates the 12-month cumulative incidence of amputation by isolating the interference of premature death, thereby generating statistically robust risk values.

Notably, the binary model candidates and competing-risk model shared identical input variables, study population, and 12-month prediction window. Therefore, any differences in risk estimates and model performance between the two approaches were solely derived from distinct statistical event definitions and analytical strategies, rather than inconsistent variable selection or study design. This standardized modeling framework ensured a fair and direct comparison between binary machine learning approaches and competing-risk regression for 12-month amputation prediction.

#### 2.7.3 SHAP interpretability

SHAP values computed via Python 3.0 visualized global feature importance and directional impacts (protective/hazardous). Individualized risk profiling enabled precise identification of high-risk patients through personalized SHAP waterfall plots.

For the competing-risk model, a quantitative nomogram was generated to provide intuitive visual scoring and convenient clinical risk assessment, forming complementary interpretability with SHAP analysis of the binary model.

#### 2.7.4 Web calculator

To facilitate convenient, real time, and visualized clinical risk stratification, a unified web-based risk calculator was developed using R software (version 4.5.2) based on the Shiny package. This web tool was constructed to integrate two analytical frameworks established in the present study, including binary machine learning modeling and competing risk regression modeling. With unified input of clinical predictor variables, the calculator is capable of generating synchronized 12-month amputation risk estimates derived from the two modeling strategies. This dual-framework web platform was designed to synthesize risk information from distinct statistical approaches and provide comprehensive evidence to support individualized clinical decision-making.

### 2.8 Statistical methods

Data pre-processing was performed prior to model construction, including outlier detection and removal of implausible values. Patients with >25% case level missing data were excluded. For variables with ≤25% missing values, multiple imputation by FCS was applied on the pooled dataset, and categorical variables were encoded.

Baseline characteristics were compared across the training, internal validation and external validation cohorts. Normality of continuous variables was assessed using the Kolmogorov–Smirnov test. Normally distributed continuous variables were compared using Student's t-test or one-way ANOVA; non-normally-distributed variables were analysed with the Mann–Whitney U test or Kruskal–Wallis H test. Categorical variables were compared using the chi-square test. A two-sided *α* = 0.05 was set as the statistical significance threshold. SPSS 27.0 was used for baseline statistical analyses.

## 3 Results

### 3.1 Clinical characteristics

A total of 1,919 patients diagnosed with LEAD and diabetes from four medical centers were enrolled in the present study (Supplementary Figure S1). The entire cohort was divided into the modeling cohort (*n* = 967), internal validation cohort (*n* = 414), and external validation cohort (*n* = 538), with a standardized 12-month follow-up window applied to all participants.

During the 12-month observational period, clinical outcomes were categorized into three mutually exclusive statuses: amputation (index event), pre-amputation death (competing event), and censored survival. The distribution of these events across the three subgroups is summarized in Table 1. Overall, 269 patients (14.0%) underwent amputation, with 12-month amputation incidences of 14.6%, 13.3%, and 13.6% in the training, internal validation, and external validation cohorts, respectively.

**Table 1:** Distribution of 12-month clinical events across training, internal validation, and external validation cohorts.

**Status**	**Total (*n* = 1,919)**	**Training cohort (*n* = 967)**	**Internal validation cohort (*n* = 414)**	**External validation cohort (*n* = 538)**	***F***	***p***
					6.916	0.140
12-month amputation event, *n* (%)	269 (14.0)	141 (14.6)	55 (13.3)	73 (13.6)		
Pre-amputation death (competing event), *n* (%)	118 (6.1)	72 (7.4)	22 (5.3)	24 (4.5)		
Censored, *n* (%)	1532 (79.8)	754 (78)	337 (81.7)	441 (82.0)		

Baseline clinical characteristics were compared across the three subgroups, and several between-group differences were observed (Supplementary Table S3). The mean age of the overall participants was 72.7 ± 9.5 years, and males accounted for 65.7% of the total population (Table 2).

**Table 2:** Characteristics of patients with lower extremity arterial disease and diabetes *n* (%)/$\left(\bar{x}\pm s\right)$.

Characteristics	Total (*n* = 1,919)	Training cohort (*n* = 967)	Internal validation cohort (*n* = 414)	External validation cohort (*n* = 538)
**Age (year)**	72.7 ± 9.5	73.2 ± 9.2	72.3 ± 9.6	72.1 ± 9.9
Sex				
Male	1,261 (65.7)	644 (66.6)	261 (63.0)	356 (66.2)
Female	658 (34.3)	323 (33.4)	153 (37.0)	182 (33.8)
**Barthel index**	68.5 ± 20.1	70.2 ± 19.8	71.9 ± 18.7	62.9 ± 20.6
Rutherford classification				
R1	69 (3.6)	35 (3.6)	12 (2.9)	22 (4.1)
R2	156 (8.1)	85 (8.8)	27 (6.5)	44 (8.2)
R3	317 (16.5)	176 (18.2)	80 (19.3)	61 (11.3)
R4	486 (25.3)	242 (25.0)	108 (26.1)	136 (25.3)
R5	829 (43.2)	412 (42.6)	182 (44.0)	235 (43.7)
R6	62 (3.2)	17 (1.8)	5 (1.2)	40 (7.4)
BMI classification				
Underweight (<18.5)	97 (5.1)	45 (4.7)	16 (3.9)	36 (6.7)
Normal (18.5–23.9)	1,288 (67.1)	659 (68.1)	300 (72.5)	329 (61.2)
Overweight (24.0–27.9)	437 (22.8)	217 (22.4)	78 (18.8)	142 (26.4)
Obesity (≥28)	97 (5.1)	133 (13.8)	20 (4.8)	31 (5.8)
Nationality				
The Han nationality	1,883 (98.1)	944 (97.6)	403 (97.3)	536 (99.6)
Ethnic minorities	36 (1.9)	23 (2.4)	11 (2.7)	2 (0.4)
Smoking				
No	930 (48.5)	460 (47.6)	209 (50.5)	261 (48.5)
Yes	989 (51.5)	507 (52.4)	205 (49.5)	277 (51.5)
Residence				
Rural or town	1,027 (53.5)	453 (46.8)	198 (47.8)	376 (69.9)
City	892 (46.5)	514 (53.2)	216 (52.2)	162 (30.1)
Financial burden				
None	21 (1.1)	8 (0.8)	3 (0.7)	10 (1.9)
Mild	473 (24.6)	224 (23.2)	107 (25.8)	142 (26.4)
Moderate	1,146 (59.7)	602 (62.3)	78 (18.8)	298 (55.4)
Severe	279 (14.5)	133 (13.8)	20 (4.8)	88 (16.4)
Wound				
No	980 (51.1)	515 (53.3)	219 (52.9)	246 (45.7)
Yes	939 (48.9)	452 (46.7)	195 (47.1)	292 (54.3)
TG				
Normal	1,165 (60.7)	626 (64.7)	259 (62.6)	280 (52.0)
High	754 (39.3)	341 (967)	155 (37.4)	258 (48.0)
TC				
Low	55 (2.9)	28 (2.9)	15 (3.6)	12 (2.2)
Normal	1,344 (70.0)	680 (70.3)	281 (67.9)	383 (71.2)
High	520 (27.1)	259 (26.8)	118 (28.5)	143 (26.6)
HDL				
Low	810 (42.2)	432 (44.7)	174 (42.0)	204 (37.9)
Normal	1,033 (53.8)	494 (51.1)	213 (51.4)	326 (60.6)
High	76 (4.0)	41 (967)	27 (6.5)	8 (1.5)
ApoA1				
Low	134 (7.0)	79 (8.2)	30 (7.2)	25 (4.6)
Normal	855 (44.6)	440 (45.5)	188 (45.4)	227 (42.2)
High	930 (48.5)	448 (46.3)	196 (47.3)	286 (53.2)
LDL				
Low	77 (4.0)	23 (2.4)	7 (1.7)	47 (8.7)
Normal	1,493 (77.8)	748 (77.4)	321 (77.5)	424 (78.8)
High	349 (18.2)	196 (20.3)	86 (20.8)	67 (12.5)
ApoB				
Low	434 (22.6)	238 (24.6)	90 (31.7)	106 (19.7)
Normal	1,280 (66.7)	635 (65.7)	278 (67.1)	367 (68.2)
High	205 (10.7)	94 (9.7)	46 (11.1)	65 (12.1)
PT				
Low	56 (2.9)	32 (3.3)	10 (2.4)	14 (2.6)
Normal	1,403 (73.1)	730 (75.5)	297 (71.7)	376 (69.9)
High	460 (24.0)	205 (21.2)	107 (25.8)	148 (27.5)
INR				
Normal	1,819 (94.8)	923 (95.4)	393 (94.9)	503 (93.5)
High	100 (5.2)	44 (4.6)	21 (5.1)	35 (6.5)
FIB				
Low	41 (2.1)	24 (2.5)	13 (3.1)	4 (0.7)
Normal	672 (35.0)	352 (36.4)	143 (34.5)	177 (32.9)
High	1,206 (62.8)	591 (61.1)	258 (62.3)	357 (66.4)
APTT				
Low	244 (12.7)	121 (12.5)	43 (10.1)	81 (15.1)
Normal	1,362 (71.0)	675 (69.8)	300 (72.5)	387 (71.9)
High	313 (16.3)	171 (17.7)	72 (17.4)	70 (13.0)
CRP				
Low	115 (6.0)	35 (3.6)	12 (2.9)	68 (12.6)
Normal	1,036 (54.0)	483 (49.9)	233 (56.3)	320 (59.5)
High	768 (40.0)	449 (46.4)	169 (40.8)	150 (27.9)
RBG				
Normal	491 (25.6)	236 (24.4)	100 (24.2)	155 (28.8)
High	1,428 (74.4)	731 (75.6)	314 (75.8)	383 (71.2)
HbA1c				
Normal	124 (6.5)	68 (7.0)	28 (6.8)	28 (5.2)
High	1,795 (93.5)	899 (93.0)	386 (93.2)	510 (94.8)
Hcy				
Normal	1,690 (88.1)	866 (89.6)	359 (86.7)	465 (86.4)
High	229 (11.9)	101 (10.4)	55 (13.3)	73 (13.6)
WBC				
Normal	1,391 (72.5)	740 (76.5)	315 (76.1)	336 (62.5)
High	528 (27.5)	227 (23.5)	99 (23.9)	202 (37.5)
PCT				
Normal	1,554 (81.0)	820 (84.8)	364 (87.9)	370 (68.8)
High	365 (19.0)	147 (15.2)	50 (12.1)	168 (31.2)
FBG				
Low	88 (4.6)	24 (2.5)	12 (2.9)	52 (9.7)
Normal	519 (27.0)	243 (25.1)	94 (22.7)	182 (33.8)
High	1,312 (68.4)	700 (72.4)	308 (74.4)	304 (56.5)
PALB				
Low	820 (42.7)	418 (43.2)	161 (38.9)	241 (44.8)
Normal	1,099 (57.3)	549 (56.8)	253 (61.1)	297 (55.2)
MONO				
Normal	1,245 (64.9)	608 (62.9)	282 (68.1)	355 (66.0)
High	674 (35.1)	359 (37.1)	132 (31.9)	183 (34.0)
NEUT				
Normal	1,200 (62.5)	660 (68.3)	263 (63.5)	277 (51.5)
High	719 (37.5)	307 (31.7)	151 (36.5)	261 (48.5)
LYM				
Low	53,427.8)	271 (28.0)	120 (29.0)	143 (26.6)
Normal	1,349 (70.3)	679 (70.2)	287 (69.3)	383 (71.2)
High	36 (1.9)	17 (1.8)	7 (1.7)	12 (2.2)
MHR				
Normal	701 (36.5)	346 (35.8)	156 (37.7)	199 (37.0)
High	1,218 (63.5)	621 (64.2)	258 (62.3)	339 (63.0)
Hypertension				
No	433 (22.6)	205 (21.2)	82 (19.8)	146 (27.1)
Yes	1,486 (77.4)	762 (78.8)	332 (80.2)	392 (72.9)
Hyperlipidemia				
No	1,591 (82.9)	781 (80.8)	319 (77.1)	491 (91.3)
Yes	328 (17.1)	186 (19.2)	95 (22.9)	47 (8.7)
CHD				
No	1,377 (71.8)	675 (69.8)	297 (71.7)	405 (75.3)
Yes	542 (28.2)	292 (30.2)	117 (28.3)	133 (24.7)
Diabetic Foot				
No	1,393 (72.6)	668 (69.1)	285 (68.8)	440 (81.8)
Yes	526 (27.4)	299 (30.9)	129 (31.2)	98 (18.2)
CKD				
No	1,619 (84.4)	792 (81.9)	321 (77.5)	506 (94.1)
Yes	300 (15.6)	175 (18.1)	93 (22.5)	32 (5.9)
DRF				
No	1,833 (95.5)	914 (94.5)	389 (94.0)	530 (98.5)
Yes	86 (4.5)	148 (15.3)	25 (6.0)	8 (1.5)
COPD				
No	1,676 (87.3)	819 (84.7)	344 (83.1)	513 (95.4)
Yes	243 (12.7)	148 (15.3)	70 (16.9)	25 (4.6)
DPN				
No	1,584 (82.5)	755 (78.1)	325 (78.5)	504 (93.7)
Yes	335 (17.5)	212 (21.9)	89 (21.5)	34 (6.3)
HF				
No	1,902 (99.1)	958 (99.1)	410 (99.0)	534 (99.3)
Yes	17 (0.9)	9 (0.9)	4 (1.0)	4 (0.7)
MI				
No	1,890 (98.5)	947 (97.9)	410 (99.0)	533 (99.1)
Yes	29 (1.5)	20 (2.1)	4 (1.0)	5 (0.9)
PCI				
No	1,725 (89.9)	863 (89.2)	369 (89.1)	493 (91.6)
Yes	194 (10.1)	104 (10.8)	45 (10.9)	45 (8.4)
CCI				
Mild	152 (7.9)	60 (6.2)	30 (7.2)	62 (11.5)
Moderate	778 (40.5)	346 (35.8)	144 (34.8)	288 (53.5)
Severe	989 (51.5)	561 (58.0)	240 (58.5)	188 (34.9)
Antiplatelet				
No	424 (22.1)	205 (21.2)	96 (23.2)	123 (22.9)
Yes	1,495 (77.9)	762 (78.8)	318 (76.8)	415 (77.1)
Rivaroxaban				
No	1,360 (70.9)	777 (80.4)	335 (80.9)	248 (46.1)
Yes	559 (29.1)	190 (19.6)	79 (19.1)	290 (53.9)
Statin				
No	748 (39.0)	418 (43.2)	190 (45.9)	140 (26.0)
Yes	1,171 (61.0)	549 (56.8)	224 (54.1)	398 (74.0)
Cilostazol				
No	1,650 (86.0)	782 (80.9)	340 (82.1)	528 (98.1)
Yes	269 (14.0)	185 (19.1)	74 (17.9)	10 (1.9)

BMI, body mass index; TG, triglyceride; TC, total cholesterol; HDL, High-density lipoprotein; ApoA1, apolipoprotein A1; LDL, Low-density lipoprotein; ApoB, apolipoprotein B; PT, prothrombin time; INR, international normalized ratio; FIB, fibrinogen; APTT, activated partial thromboplastin time; CRP, C-reactive protein; RBG, random blood glucose; HbA1c, hemoglobin A1c; Hcy, homocysteine; WBC, white blood cell; PCT, procalcitonin; FBG, fasting blood glucose; PALB, prealbumin; MONO, monocyte; NEUT, neutrophil; LYM, lymphocyte; MHR, monocyte to HDL cholesterol ratio(MONO/HDL); CHD, coronary heart disease; CKD, chronic kidney disease; COPD, chronic obstructive pulmonary disease; DRF, dialysis-dependent renal failure; DPN, diabetic peripheral neuropathy; HF, heart failure; MI, myocardial infarction; PCI, percutaneous coronary intervention; CCI, charlson comorbidity index.

Baseline characteristics were further compared between the overall cohort and patients lost to 12-month follow-up (Supplementary Table S4). Statistically significant differences were observed for most baseline variables, indicating that patients who were lost to follow-up were not randomly distributed across the study population. This suggested potential selection bias introduced by loss of follow-up.

### 3.2 Selection of predictors

We initially analyzed 48 candidate variables for the amputation prediction model (Supplementary Table S5). Three complementary algorithms, including LASSO logistic regression, random forest classifier, and Boruta algorithm, were applied for feature selection exclusively within the training cohort (*n* = 967).

For LASSO logistic regression, the optimal regularization parameter *λ* was determined by five-fold cross-validation, and the one-standard-error rule was adopted to obtain a parsimonious model. This procedure yielded 16 candidate predictors. Figure 1A shows the coefficient trajectory of LASSO regression, and Figure 1B illustrates variable selection under different regularization penalty strengths.

**Figure 1:**
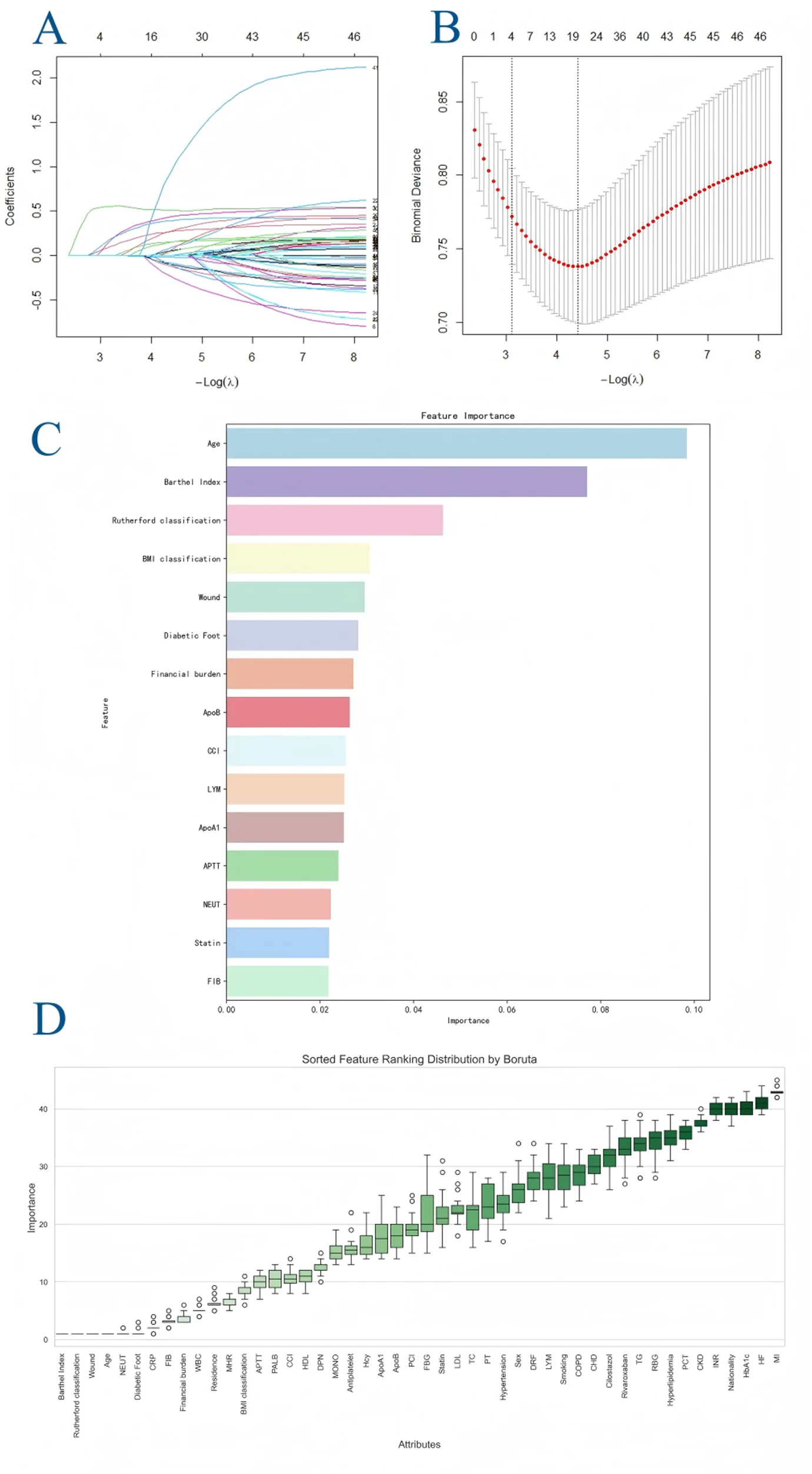
**(A)** The change characteristics of variable coefficients. **(B)** The process of selecting the optimal value of the parameter *λ* in the LASSO regression model is carried out through the cross-validation method. **(C)** Variable importance ranking of random forest method. **(D)** Sorted feature ranking distribution by the Boruta algorithm, illustrating the stability of feature importance across iterations.

The random forest classifier was utilized to compute feature-importance scores, and the top-ranked 15 variables were retained according to importance ranking (Figure 1C).

The Boruta algorithm was repeated 20 times with different random seeds to reduce stochastic screening bias; each run was configured with a maximum iteration of 50. Features consistently confirmed across repeated runs were identified as Boruta-selected predictors, and 15 variables were finally obtained (Figure 1D).

Predictors identified by at least two of the three screening algorithms were retained as the final predictive features. This consensus-based filtering procedure yielded 14 predictors (Supplementary Table S6). These predictors included Rutherford classification, financial burden, age, Barthel Index, diabetic foot, statin, fibrinogen (FIB), neutrophil (NEUT), residence, prealbumin (PALB), C-reactive protein (CRP), activated partial thromboplastin time (APTT), BMI classification and wound. The same set of 14 predictors was subsequently adopted for both binary machine-learning modeling and competing-risk regression modeling.

### 3.3 Machine learning models

Ten machine-learning algorithms including RF, LR, DT, NB, ANN, KNN, XGBoost, GraBoost, CatBoost, and LGBM were trained with hyperparameter tuning via stratified five-fold cross-validation. Predictive performance was assessed using multiple metrics: AUC, accuracy, precision, recall, F1-score, specificity, log-loss, MCC, and Brier score, alongside graphical evaluation via ROC curves and calibration curves.

In the internal validation cohort, the GraBoost model achieved the highest AUC of 0.860, accompanied by accuracy = 0.787, precision = 0.801, recall = 0.763, F1-score = 0.782, specificity = 0.811, log-loss = 0.481, MCC = 0.574, and the lowest Brier score of 0.156 among all ten models, reflecting favorable discrimination and calibration capacity. Full performance metrics for internal validation are summarized in Table 3. Supplementary Figure S2 shows ROC curves and calibration plots of the ten machine-learning algorithms in the internal validation set, and Figure 2 presents the overlaid comparative ROC curves across models for the internal validation cohort.

**Table 3:** Internal validation indicators for 10 machine learning models.

Model	AUC	Accuracy	Precision	Recall	F1-score	Specificity	Brier	Log Loss	MCC
RF	0.812	0.721	0.824	0.563	0.669	0.880	0.189	0.550	0.467
LR	0.855	0.765	0.745	0.805	0.774	0.724	0.157	0.486	0.531
DT	0.711	0.705	0.716	0.680	0.697	0.730	0.245	4.090	0.410
NB	0.834	0.728	0.670	0.900	0.768	0.557	0.178	0.525	0.486
ANN	0.823	0.747	0.806	0.649	0.719	0.844	0.177	0.542	0.503
KNN	0.673	0.646	0.687	0.538	0.603	0.755	0.291	7.295	0.300
XGBoost	0.788	0.699	0.794	0.538	0.641	0.861	0.221	0.730	0.421
GraBoost	0.860	0.787	0.801	0.763	0.782	0.811	0.156	0.481	0.574
CatBoost	0.838	0.741	0.829	0.607	0.701	0.875	0.182	0.569	0.500
LGBM	0.792	0.710	0.811	0.549	0.654	0.872	0.218	0.736	0.444

RF, random forest; LR, logistic regression; DT, decision tree; NB, naive bayes; ANN, artificial neural network; KNN, K-Nearest neighbors; XGBoost, eXtreme gradient boosting; GraBoost, gradient boosting; CatBoost, gradient boosting with categorical features support; LGBM, light gradient boosting machine; AUC, area under the curve; MCC, matthews correlation coefficient.

**Figure 2:**
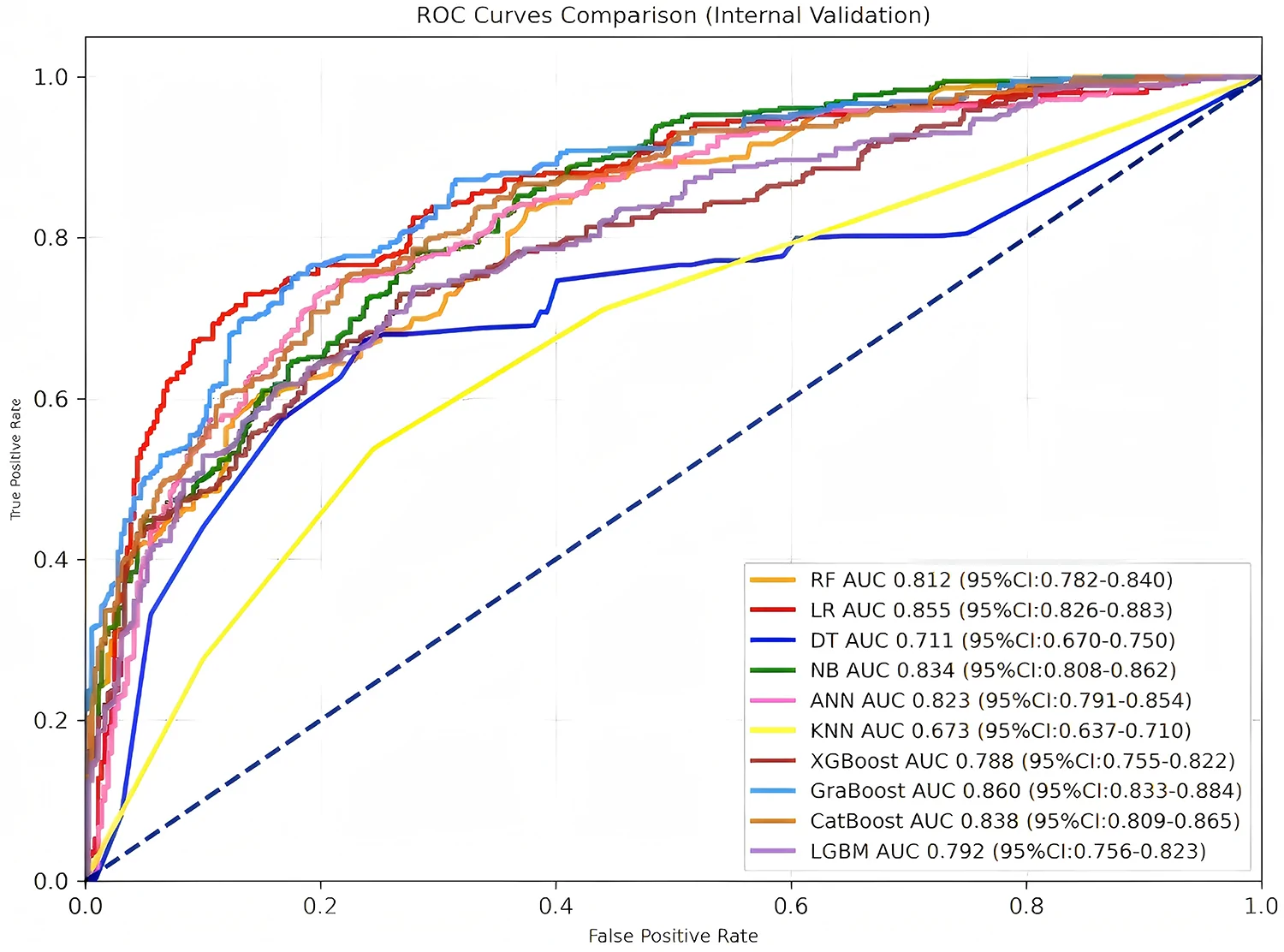
Comparison of ROC curves in internal validation for different machine learning models.

When tested in the geographically independent external validation cohort, eight models (RF, LR, NB, ANN, XGBoost, GraBoost, CatBoost, LGBM) yielded AUC values above 0.70, indicating acceptable generalizability, whereas DT and KNN showed relatively low discriminative power. Complete external validation performance metrics are provided in Table 4. Supplementary Figure S3 displays corresponding ROC and calibration curves for external validation, and Figure 3 superimposes ROC trajectories for cross model comparison in the external validation cohort.

**Table 4:** External validation indicators for 10 machine learning models.

Model	AUC	Accuracy	Precision	Recall	F1-score	Specificity	Brier	Log Loss	MCC
RF	0.810	0.690	0.772	0.540	0.635	0.841	0.191	0.559	0.399
LR	0.788	0.747	0.724	0.800	0.760	0.695	0.186	0.552	0.497
DT	0.722	0.710	0.702	0.729	0.715	0.690	0.218	2.488	0.420
NB	0.785	0.713	0.705	0.733	0.719	0.692	0.202	0.644	0.426
ANN	0.760	0.625	0.712	0.419	0.528	0.830	0.220	0.621	0.274
KNN	0.586	0.556	0.570	0.456	0.507	0.656	0.346	8.177	0.114
XGBoost	0.782	0.637	0.736	0.426	0.540	0.847	0.247	0.756	0.301
GraBoost	0.828	0.760	0.800	0.695	0.743	0.826	0.178	0.540	0.525
CatBoost	0.795	0.683	0.764	0.529	0.625	0.837	0.207	0.611	0.384
LGBM	0.795	0.669	0.775	0.475	0.589	0.862	0.221	0.670	0.366

RF, random forest; LR, logistic regression; DT, decision tree; NB, naive bayes; ANN, artificial neural network; KNN, K-Nearest neighbors; XGBoost, eXtreme gradient boosting; GraBoost, gradient boosting; CatBoost, gradient boosting with categorical features support; LGBM, light gradient boosting machine; AUC, area under the curve; MCC, matthews correlation coefficient.

**Figure 3:**
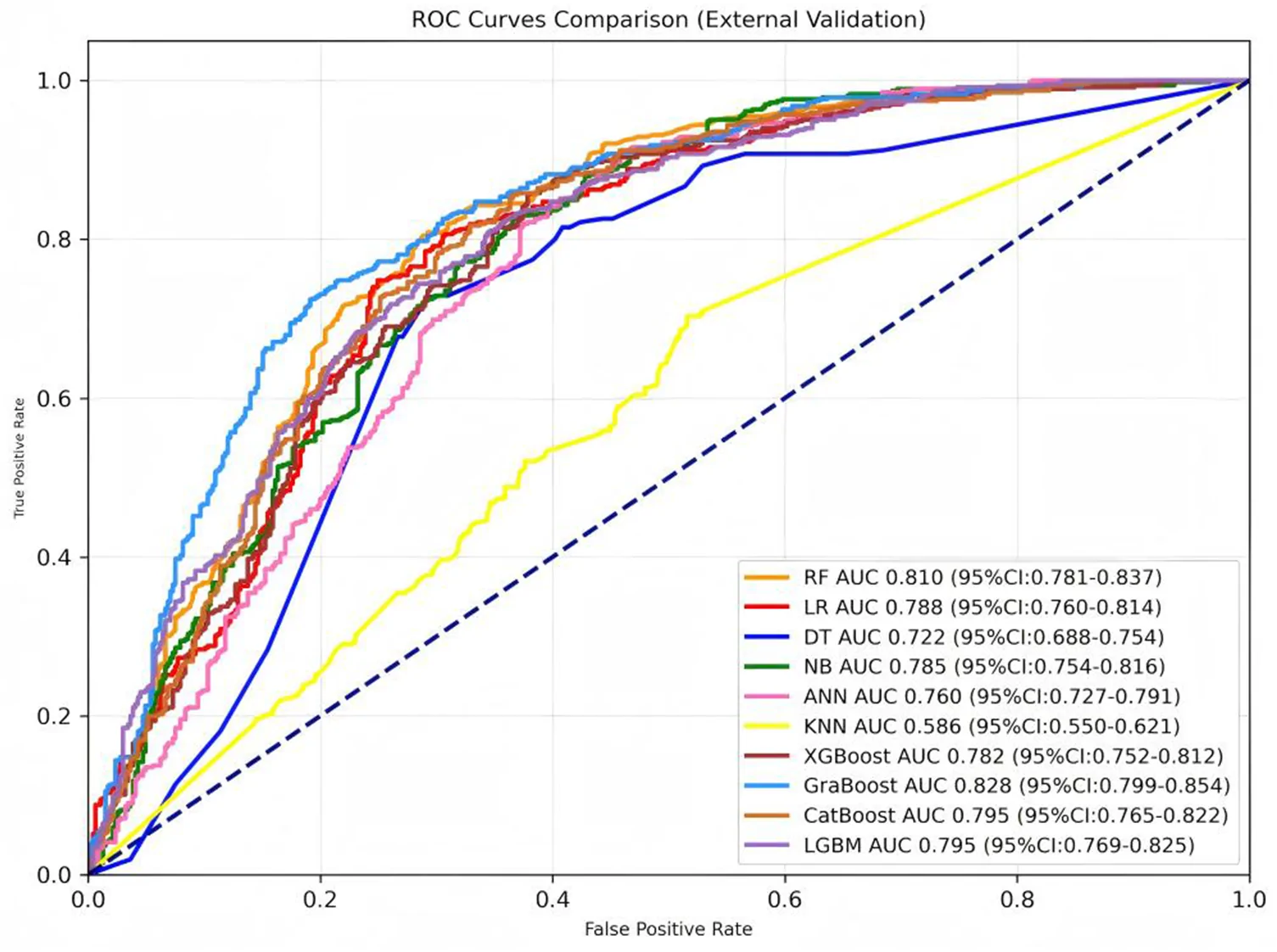
Comparison of ROC curves external validation for different machine learning.

The GraBoost model was designated as the final optimal model based on the comprehensive integration of internal and external validation performance. In internal validation, GraBoost attained an AUC of 0.860, accuracy = 0.787, precision = 0.801, F1-score = 0.782, MCC = 0.574, specificity = 0.811, and log-loss = 0.481. DeLong's test was conducted to compare the AUC of GraBoost against the other nine machine-learning models (Supplementary Table S7). Statistically significant differences in AUC were observed between GraBoost and most competing models. Although several boosting-based algorithms (XGBoost, CatBoost, LGBM) had comparable AUC, GraBoost produced the minimal Brier score, indicating better probabilistic calibration (Supplementary Figure S4). In external validation, GraBoost maintained competitive performance with AUC = 0.828, accuracy = 0.760, precision = 0.800, F1-score = 0.695, MCC = 0.525, specificity = 0.826, and log-loss = 0.540. Compared with CatBoost and LGBM, GraBoost exhibited more balanced overall metrics and avoided the extreme performance fluctuations seen in the KNN model. Furthermore, GraBoost showed relatively limited performance degradation from internal to external validation, supporting its anti-overfitting property. According to DeLong's test results for the external validation cohort (Supplementary Table S7), some models did not show statistically significant AUC differences vs. GraBoost; nevertheless, GraBoost achieved more favorable composite performance across key discrimination and calibration indicators.

Collectively, these results supported GraBoost as the preferred predictive model for 12-month amputation risk among patients with LEAD and diabetes.

### 3.4 Fine-Gray competing-risk model

Using the identical set of 14 predictors identified in the feature selection step, a Fine-Gray competing-risk regression model was constructed within the training cohort (*n* = 967). 12-month amputation was defined as the index event, and pre-amputation all-cause death was treated as the competing event.

Time-dependent AUC calculated at the 12-month time-point was 0.762 in the training cohort, 0.711 in the internal-validation cohort (*n* = 414), and 0.811 in the external-validation cohort (*n* = 538) (Figure 4A). The 12-month Brier scores were 0.092 for the training cohort, 0.106 for the internal-validation cohort, and 0.085 for the external-validation cohort. Calibration curves for 3-month, 6-month, and 12-month endpoints illustrated the agreement between predicted cumulative incidence and observed amputation probability across risk strata (Figure 4B).

**Figure 4:**
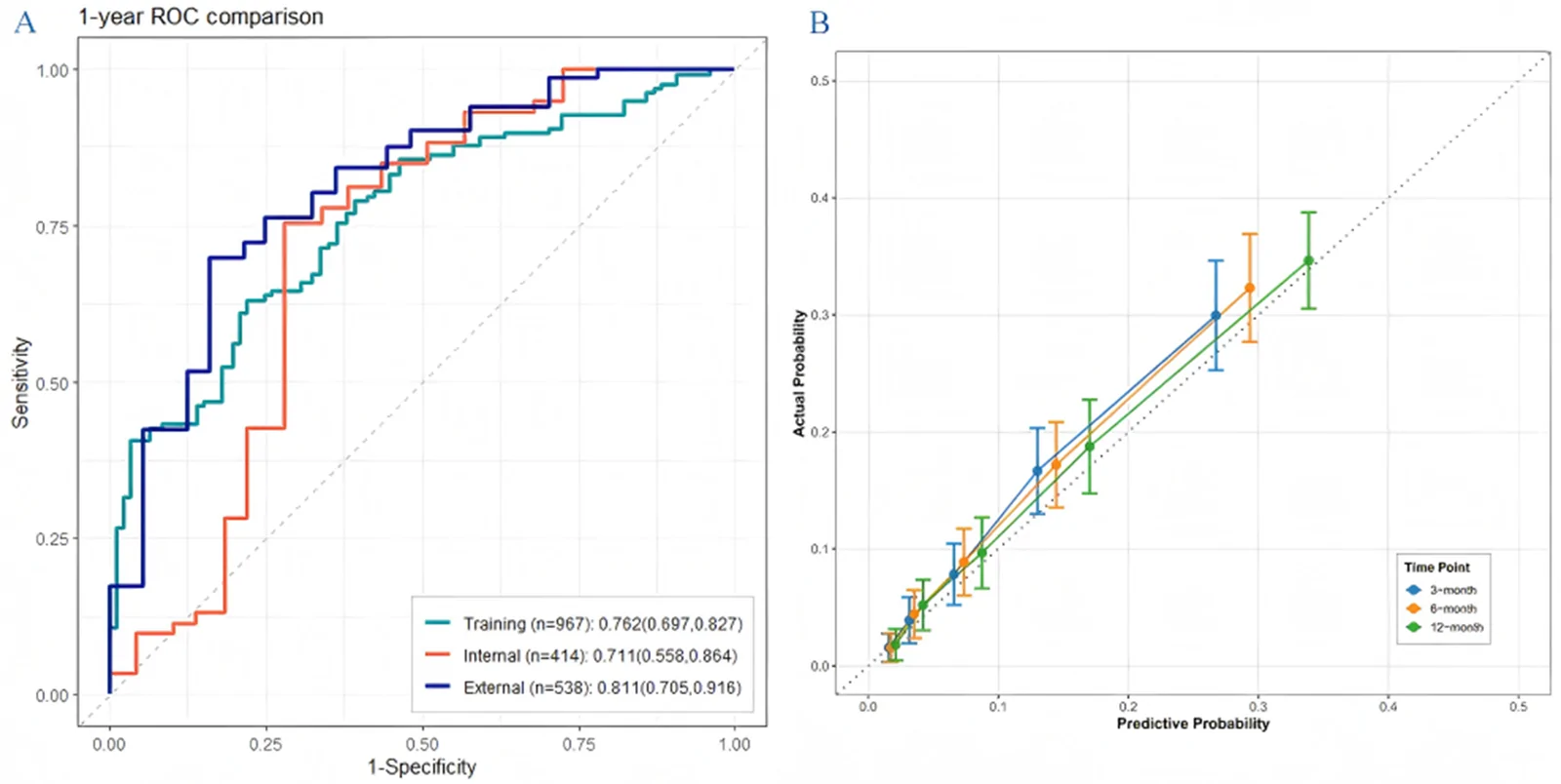
**(A)** Time-dependent receiver operating characteristic (ROC) curves for 12-month amputation prediction in the training, internal validation, and external validation cohorts. The *x*-axis shows 1-specificity, and the *y*-axis represents sensitivity. The area under the ROC curve (AUC) values with 95% confidence intervals are presented in the legend for the training cohort (*n* = 967), internal validation cohort (*n* = 414), and external validation cohort (*n* = 538). **(B)** The *x*-axis represents the model-predicted probability of amputation, and the *y*-axis denotes the observed actual amputation probability. The dotted diagonal line indicates perfect calibration. Colored points with error bars show the observed probability within each risk bin at 3-month (blue), 6-month (orange), and 12-month (green) time points.

Cumulative incidence function (CIF) curves for amputation across the three cohorts are presented in Figure 5. Gray's test demonstrated no significant difference in 12-month amputation cumulative incidence among training, internal validation, and external validation cohorts (*p* = 0.722). A nomogram derived from the Fine-Gray model was generated to obtain individual-level 3-month, 6-month and 12-month predicted cumulative amputation incidence (Figure 6).

**Figure 5:**
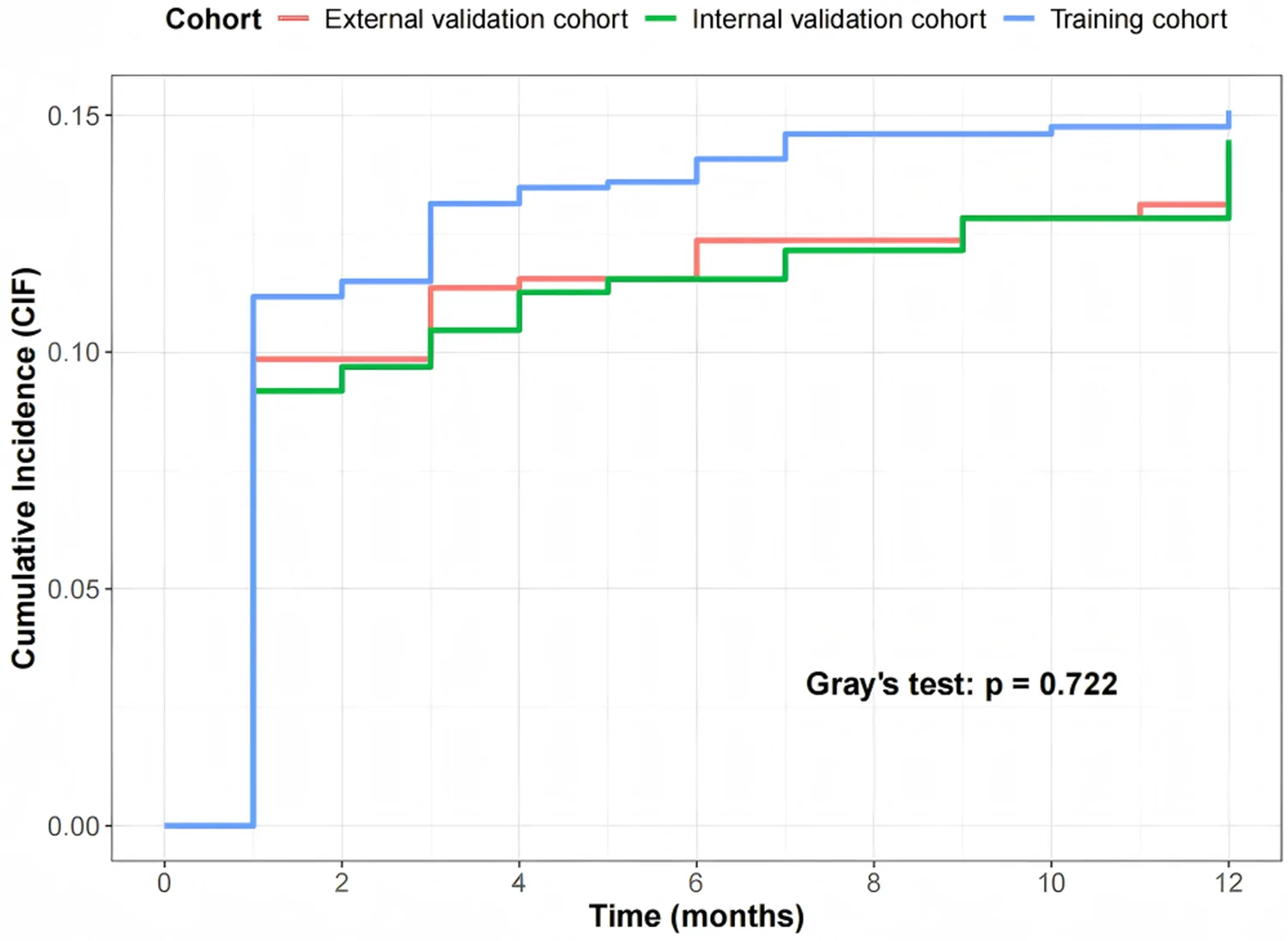
Cumulative incidence function (CIF) curves of amputation for training (blue), internal validation (green), and external validation (red) cohorts over the 12-month follow-up period, accounting for death as the competing event. Gray's test was used to compare between-group differences in cumulative amputation incidence, with *p* = 0.722.

**Figure 6:**
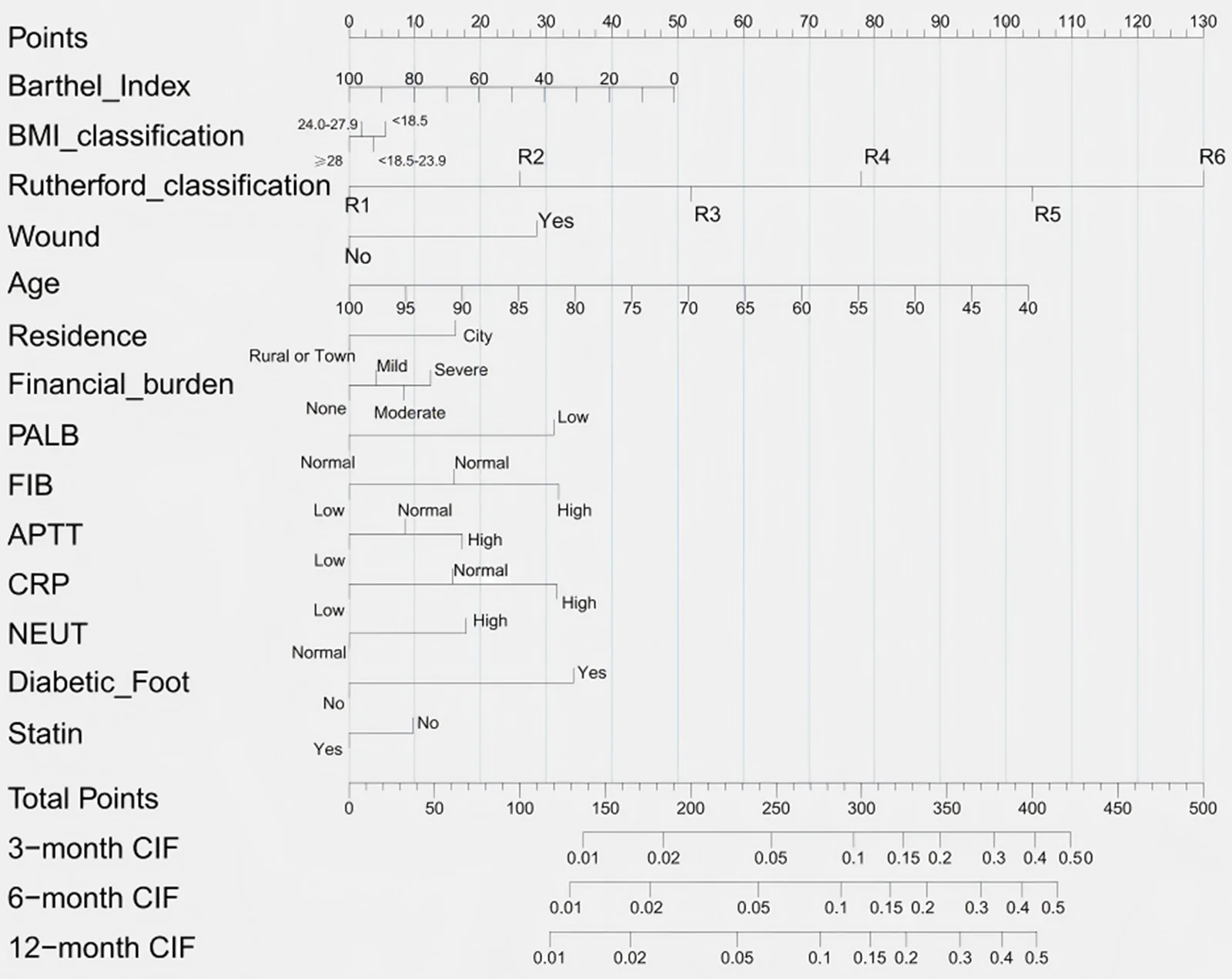
This nomogram was constructed using the 14 predictors retained in the Fine-Gray competing-risk model, where death before amputation was defined as the competing event. To estimate an individual patient's 12-month amputation cumulative incidence, the points corresponding to each predictor value are summed to obtain the total points, and the predicted probability of 12-month amputation is read from the bottom axis.

### 3.5 Variable importance and variable interpretation

The SHAP interpretability framework was employed to deconstruct the predictive mechanics of the GraBoost model and quantify feature importance hierarchies. Figure 7A demonstrates the significant discriminative capacity of all variables in distinguishing relevant factors in the training validation cohort, which validates their clinical utility. Rutherford classification exhibited the strongest predictive influence, followed by financial burden and age. Barthel index, diabetic foot, statin, FIB, NEUT, residence, and PALB demonstrated moderate effects, while CRP, APTT, BMI classification and wound showed comparatively weaker associations. In addition, the SHAP dependence plot (Figure 7B) illustrates feature value SHAP value relationships. Advanced Rutherford classification, lower Barthel Index, and younger age were significantly associated with increased amputation risk. The presence of wounds and diabetic foot showed a positive association with risk. Conversely, urban residence, lower financial burden, normal BMI range and statin use were correlated with a reduced amputation rate. Among laboratory parameters, elevated levels of FIB, APTT, CRP, and NEUT were associated with increased amputation risk. In contrast, a higher level of PALB served as a protective factor against amputation. To elucidate feature level impact mechanisms on individual predictions and assess clinical translatability, a representative case was selected from the cohort for SHAP-based interpretability analysis. Figure 7C visually demonstrates GraBoost's prediction mechanics at the individual patient level. SHAP values in our model quantify directional contributions of patient specific features to amputation risk predictions.

**Figure 7:**
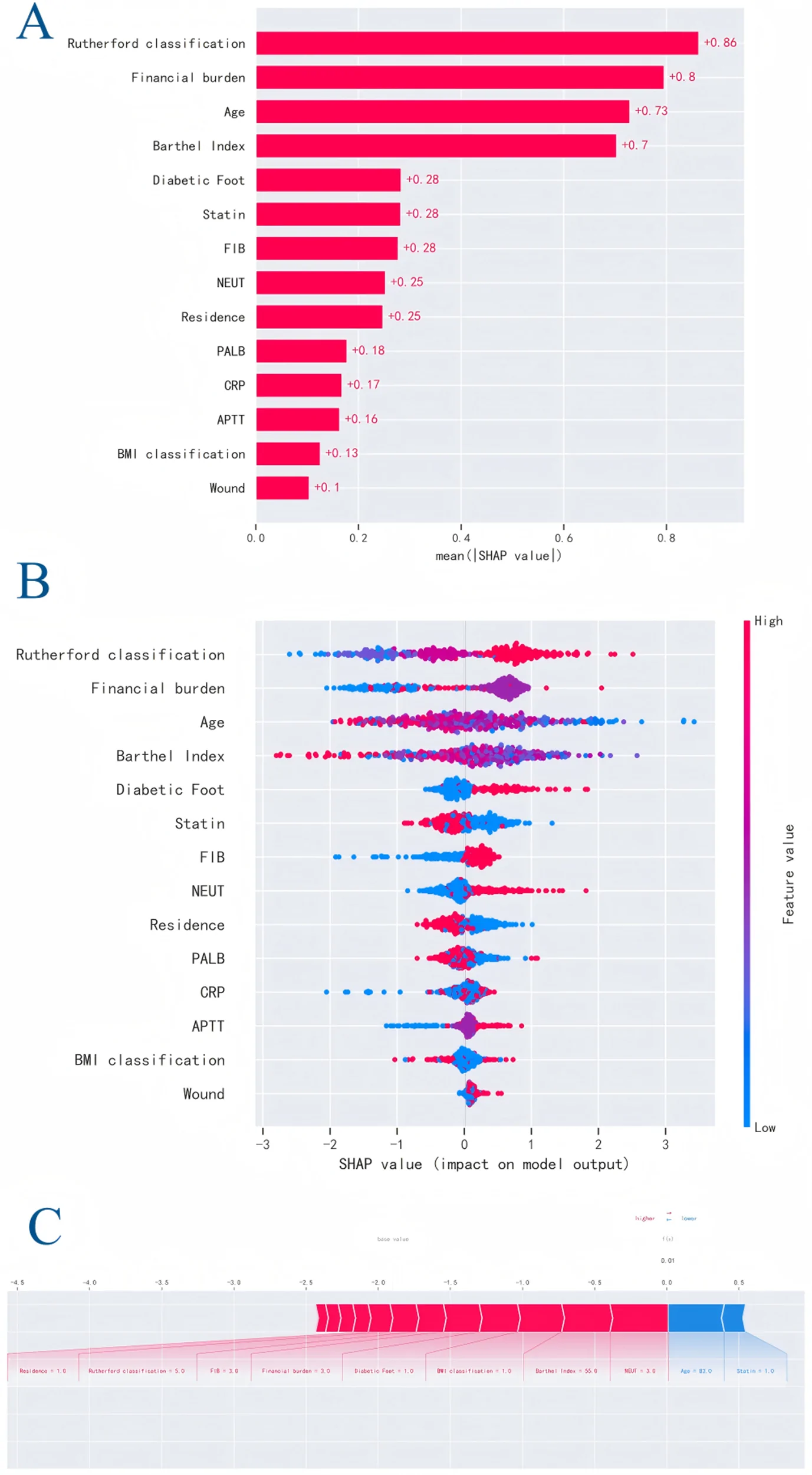
**(A)** Illustrates the feature importance ranking. **(B)** Illustrates the feature contribution patterns through a SHAP bee swarm plot. With red/blue markers indicating elevated/diminished feature magnitudes respectively. The *x*-axis represents SHAP values derived from feature scales, where positive values denote increased amputation risk and negative values suggest risk mitigation. Feature importance hierarchies were established through SHAP absolute value ranking across the entire cohort. **(C)** Provides an individualized prediction explanation using a SHAP force plot.

A nomogram was constructed based on the Fine-Gray competing risk regression model (Figure 6). Each predictor variable including Barthel Index, BMI classification, Rutherford classification, wound status, age, residence, financial burden, PALB, FIB, APTT, CRP, NEUT, diabetic foot, and statin use was assigned a corresponding point score according to the model regression coefficients. Individual total points were summed from the point value of each variable. By locating the total points scale, readers could obtain the corresponding predicted 3-month, 6-month and 12-month cumulative incidence of amputation under the competing risk framework.

Both the Fine-Gray competing risk model and the binary GraBoost model were built on the same 14 predictor set with a unified 12-month prediction horizon.

### 3.6 Implementation of web calculator

Both the binary GraBoost model and the Fine-Gray competing risk regression model for predicting 12-month amputation risk in patients with LEAD and diabetes were integrated and deployed on a Shiny web-based platform. The web-based risk prediction calculator is available at: https://tanglu.shinyapps.io/AmputationRisk/.

The calculator interface consists of a left-side parameter input panel, with dropdown menus for categorical predictors and slider widgets for continuous variables. After entering patient specific clinical parameters and triggering the “Calculate Risk” function, real-time risk outputs from the two distinct prediction frameworks are displayed in the right-hand output panel. Illustrative examples including high-risk patient prediction scenarios are provided as practical operation references (Supplementary Figure S5).

The SHAP force plot conceptualizes Shapley values as directional vectors exerting positive or negative “forces” on baseline predictions. Boldfaced log-odds values (*f* (*x*)) exceeding baseline indicate elevated amputation risk, while sub-baseline values suggest risk mitigation. The *f* (*x*) output represents case-specific log-odds ratios derived from model computations. Crimson vectors denote risk-enhancing features, whereas azure vectors reflect protective factors attenuating amputation risk. Vector magnitudes correspond to feature contribution intensities, with extended lengths indicating greater predictive influence.

## 4 Discussion

### 4.1 Amputation rates

In our overall cohort of patients with LEAD and diabetes, the 12-month amputation rate was 14.0%. This figure falls within the previously-reported range of 13%–48% for comparable patient populations (9, 10). Variations in amputation rates across published studies may stem from differences in patient inclusion criteria, disease severity distributions, healthcare systems, and follow-up definitions. Our observed amputation rate is broadly consistent with existing epidemiological findings for patients with LEAD complicated by diabetes.

### 4.2 Comparison of our model with existing models

Existing prediction models for amputation risk are mostly derived from cohorts of patients with diabetic foot (30, 38–42) or LEAD (26, 43–45). Several recent models have focused on re-amputation risk among patients with LEAD and/or diabetes (46, 47). Nevertheless, few externally validated prediction tools are specifically established for patients with concurrent LEAD and diabetes. Most previously published amputation risk models rely on Cox proportional hazards regression. Compared with conventional regression approaches, gradient boosting based machine learning algorithms such as GraBoost can capture complex nonlinear relationships within clinical datasets while mitigating overfitting, and have seen increasing use in clinical predictive modelling research (48).

Li et al. reported an XGBoost model for predicting 3-year major adverse limb events in patients with LEAD, which achieved a high AUC of 0.880 (25). However, that model required novel biomarkers that are not routinely available in primary care environments, restricting its real world implementability. Furthermore, many existing predictive models lack rigorous external validation, model interpretability assessment, and ready to use clinical application tools, which constrains their practical clinical translation.

In the present study, we first performed a literature review to collect 75 candidate risk factors for amputation (Supplementary Table S1). After filtering according to clinical accessibility and data completeness, 48 predictors entered the subsequent analytical pipeline (Supplementary Table S2). Three complementary feature selection approaches including LASSO logistic regression, random forest classifier, and Boruta algorithm were applied within the training cohort. Predictors identified by at least two of the three algorithms were retained, yielding 14 consensus predictors (Supplementary Table S6). Ten machine learning algorithms were then trained and evaluated via internal and external validation. Among them, the GraBoost model showed relatively favourable discrimination and calibration performance. Its performance in the external validation cohort further supported acceptable stability and generalizability.

In addition to the binary GraBoost machine learning model, we further developed a Fine-Gray competing risk regression model to address pre-amputation mortality, a competing event that is rarely handled in most existing amputation risk prediction studies. Unlike conventional logistic or Cox models that treat pre-amputation death simply as a non-event, the Fine-Gray model estimates the sub-distribution cumulative incidence of amputation, which better reflects real world clinical scenarios where patients may die before amputation occurs.

The final GraBoost model incorporated 14 readily available clinical variables: Rutherford classification, financial burden, age, Barthel Index, diabetic foot, statin use, FIB, NEUT, residence, PALB, CRP, APTT, BMI classification and wound. All predictors represent routine clinical indicators widely measurable across different tiers of hospitals, supporting practical feasibility. To facilitate understanding of model outputs, SHAP analysis was implemented for model interpretation. Moreover, we constructed a publicly accessible web-based calculator embedding both the GraBoost and Fine-Gray competing risk models, allowing clinicians to obtain dual risk estimates and support corresponding clinical management decisions.

### 4.3 Explanation of findings

#### 4.3.1 Vascular function and self-care capacity

Our analysis identified the Rutherford classification and Barthel Index as core predictors within the model. A higher Rutherford classification and a lower Barthel Index were associated with an increased risk of amputation. The Rutherford classification serves as the gold-standard tool for assessing the severity of LEAD in clinical practice (49). In patients with LEAD and diabetes, the Rutherford classification emerges as a critical prognostic indicator, with higher grades signifying an exponentially increased probability of amputation. The Rutherford classification (R0-R6) (50–53) stratifies disease severity, with ascending grades reflecting progressive clinical deterioration. The critical thresholds are at Rutherford classification R4 (rest pain), R5 (tissue loss), and R6 (major tissue loss), which indicate severe limb ischemia and a marked increase in risk in diabetic populations. In patients with diabetes, concurrent neuropathy and heightened susceptibility to infection accelerate disease progression and amplify the risk of amputation (54, 55). Low self-care ability is not merely a consequence of disease severity but acts as an “amplifier” that actively exacerbates the condition, creating a vicious cycle (56). A lower Barthel Index, reflecting increased dependency in activities of daily living, signifies that the patient is caught in a vicious cycle where functional decline, impaired self-management, and local pathology mutually reinforce each other (57, 58). The lack of self-care capacity leads to the failure in timely detection and management of minor foot trauma. Limited mobility exacerbates disuse muscle atrophy and local hypoperfusion in the lower limbs. The impaired systemic self-management of chronic disease that typically accompanies a low Barthel Index further compromises tissue repair and anti-infective capacity (59). These factors increase the patient's risk of amputation.

#### 4.3.2 Sociodemographic characteristics

Previous studies have indicated that older age is associated with a higher risk of developing arteriosclerosis obliterans of the lower extremities and subsequent amputation (60–65). Interestingly, within our cohort of patients already diagnosed with concomitant LEAD and diabetes, both the GraBoost machine-learning model and Fine-Gray competing-risk regression model identified that younger age was associated with elevated 12-month amputation risk. Since Fine-Gray sub-distribution hazard ratios are statistically adjusted for pre-amputation death as a competing event, this finding cannot be fully attributed to older patients dying prior to limb-loss events. Several potential explanations may account for this counter-intuitive observation in patients with established disease. First, age related differences in clinical treatment decision making may play a role. For elderly patients with severe limb ischaemia, clinicians may prefer conservative palliative care and avoid major amputation given their limited life expectancy. By contrast, younger patients are more likely to receive aggressive surgical intervention including amputation once severe limb complications occur. Second, younger patients who progress to severe limb threatening ischaemia might represent a more biologically aggressive disease phenotype: diabetes related vasculopathy may progress more rapidly in this subgroup. Third, unmeasured residual confounding cannot be excluded in this retrospective real-world cohort. These interpretations remain speculative and require further validation in prospective multicenter datasets.

Our two models also indicated that rural town residence and greater financial burden were associated with increased amputation risk. Possible underlying explanations include limited access to medical resources, socioeconomic disadvantage, delayed diagnosis, deferred intervention, and suboptimal treatment adherence among these patient groups (62, 66–69). Moreover, a U-shaped association between BMI and amputation risk was observed: both underweight and elevated BMI corresponded to higher amputation risk. This is consistent with previous studies (70). Underweight status may reflect malnutrition, sarcopenia and severe underlying disease burden, whereas high BMI related metabolic disturbance, chronic inflammation and peripheral metabolic dysfunction could collectively contribute to worse limb outcomes.

#### 4.3.3 Comorbidities

The presence of a wound (including both arterial and venous ulcers, as well as other wound types) or diabetic foot significantly increases the amputation risk in patients with comorbid LEAD and diabetes. These factors are reflective of tissue ischemia and can exacerbate the ischemic process, leading to infection and impaired healing (71). Firstly, the presence of a wound, whether arterial ischemic or venous stasis ulcer, is exceptionally difficult to heal and highly prone to secondary infection in the setting of perfusion deficit caused by LEAD. Infection further aggravates tissue necrosis and ischemia, creating a vicious cycle that directly leads to amputation (72, 73). Secondly, diabetic foot is a complex clinical syndrome resulting from the interplay of multiple factors including neuropathy, ischemia, and infection. Diabetic foot itself is frequently complicated by LEAD, and its associated amputation risk is 4 times higher than that of LEAD or diabetes alone (67, 87).

#### 4.3.4 Medication therapy

Statin use is inversely correlated with amputation risk. Users have a lower incidence of amputation than non-users, which may be due to plaque stabilization, anti-inflammatory effects, and LDL-C-dependent vascular repair mechanisms (74–76). Statins protect against limb loss and cardiovascular events in LEAD patients, reducing the risks of amputation, cardiovascular mortality, and all-cause mortality. Patients with end-stage renal disease and LEAD receiving statin therapy have lower amputation rates and mortality compared with non-users (77). Among patients with chronic limb-threatening ischemia, statin use improves 1-year limb patency rates and reduces the risks of amputation (78) and reintervention (79). Lipid-lowering agents improve walking distance, claudication symptoms, and pain-free walking time (80–82, 88).

#### 4.3.5 Biomarkers

The biomarkers included in our study primarily encompassed coagulation parameters (FIB, APTT), inflammation-related markers (CRP, NEUT, PALB).

In patients with LEAD and diabetes, elevated levels of FIB and APTT were significant risk factors for predicting amputation. A high FIB level reflects a hypercoagulable state and persistent inflammation, which promote atherosclerosis progression, thrombosis, and compromise vascular endothelial function (56). This consequently affects post-revascularization vessel patency and tissue perfusion, ultimately increasing amputation risk. Prolonged APTT in this population often does not merely indicate enhanced anticoagulation but is more likely to reflect a coagulation imbalance due to impaired hepatic synthetic function, consumption of vitamin K-dependent clotting factors, among others (82). This imbalance further impairs microcirculatory perfusion in the lower limbs and hinders effective healing of ulcerated wounds. Acting synergistically with diabetic vascular endothelialopathy, it ultimately renders the ischemic limb more susceptible to progressive gangrene and uncontrolled infection, significantly increasing the risk of limb salvage failure.

Atherosclerosis, the core pathological basis of LEAD, is essentially a chronic inflammatory response (86). In patients with diabetes, the hyperglycemic state exacerbates systemic chronic low-grade inflammation. This inflammatory state increases amputation risk through several mechanisms (83). (1) Promoting disease progression: The inflammatory response accelerates the formation and destabilization of atherosclerotic plaques and vascular endothelial dysfunction, leading to more severe and extensive lower limb ischemia. (2) Affecting treatment outcomes: Multiple studies have shown that elevated inflammatory markers are significantly associated with post-revascularization restenosis, limb salvage failure, and increased amputation risk (84, 85). (3) Synergistic worsening with infection: Diabetic patients inherently have immune dysregulation and delayed wound healing. When lower limb ischemia is complicated by infection, it triggers a more intense systemic inflammatory response, forming a vicious cycle that greatly increases the likelihood of tissue necrosis and the eventual need for amputation. CRP is a marker reflecting the level of systemic inflammation; NEUT, as key inflammatory cells, has its elevated count directly reflecting intensified systemic inflammatory response; a decreased PALB level is a sensitive indicator of inflammatory activity in the body. Therefore, elevated CRP and NEUT, along with decreased PALB, are associated with an increased risk of amputation (90).

### 4.4 Clinical implications

In clinical practice, the dual-model web calculator can be used at the point of care in vascular surgery, endocrinology, and diabetic-foot clinics to estimate the 12-month amputation risk of individual patients with LEAD and diabetes. Patients identified as high risk can be prioritized for intensified foot surveillance, timely revascularization, multidisciplinary foot-care input, and optimization of cardiovascular risk-factor management (89).

### 4.5 Limitation

Several limitations of this study should be acknowledged when interpreting our findings. First, this was a retrospective multicenter observational study. Despite strict data preprocessing, inherent selection bias and incomplete clinical data cannot be fully ruled out, and the performance of our dual-model prediction system requires further validation in prospective independent cohorts. Second, partial imbalances in baseline characteristics existed among the training, internal validation and external validation cohorts. Although Gray's test showed comparable 12-month amputation cumulative incidence across groups, baseline disparities may still affect model generalizability.

The relatively small sample size of the internal validation cohort led to wide confidence intervals for the time-dependent AUC of the Fine-Gray model, bringing uncertainty to the estimation of its discriminative ability. In addition, all participants were Chinese patients with LEAD and diabetes; thus caution is needed when extrapolating these models to other ethnic populations or different healthcare contexts, and our models were developed exclusively for 12-month amputation risk assessment and are not applicable for longer-term outcome prediction.

Notably, multiple imputation using the fully conditional specification was performed on the pooled entire dataset prior to partitioning into training and validation cohorts, which may introduce a degree of data leakage. Although feature selection was strictly restricted to the training cohort to mitigate this risk, this workflow remains an important limitation. Furthermore, the web-based calculator embedding both GraBoost and Fine-Gray models has not been prospectively validated in real world clinical settings; it serves only as an auxiliary risk stratification tool and cannot replace comprehensive clinical judgment. Some counter intuitive associations such as higher amputation risk among relatively younger patients were observed, and potential mechanisms proposed in the discussion remain speculative, as causal inference cannot be drawn from retrospective observational data. Finally, significant differences in most baseline characteristics were detected between patients lost to 12-month follow-up and the overall cohort, implying non-random loss to follow-up that may introduce selection bias and impair the robustness of risk estimation results.

## 5 Conclusion

This study developed and validated two complementary prediction frameworks: the interpretable machine learning based GraBoost model and the Fine-Gray competing risk regression model for predicting 12-month amputation risk in patients with LEAD and diabetes. A unified web-based calculator embedding both models was deployed. The final models incorporated 14 predictors: Rutherford classification, financial burden, age, Barthel Index, diabetic foot, statin, FIB, NEUT, residence, PALB, CRP, APTT, BMI classification and wound. This framework enables quantitative risk estimation and assists clinicians in stratifying high-risk patients to guide clinical management.

## Data Availability

The raw data supporting the conclusions of this article will be made available by the authors, without undue reservation.
