# Supplementary material for: Development and validation of a machine learning-based model for predicting amputation risk in patients with lower extremity arterial disease and diabetes
Source: Front Cardiovasc Med. 2026 Oct 20;13:1893875. doi: 10.3389/fcvm.2026.1893875 (PMC13727486; doi:10.3389/fcvm.2026.1893875)
Supplement: Supplementary file 1 [file Datasheet1.docx]

**Supplemental Figure 1 Analytical sample flow chart**

**Supplemental Figure 2 ROC curves and calibration curves in internal validation of different machine learning models**

Abbreviation:A represents for random forest (RF) internal validation ROC curve, B represents for RF internal validation calibration curve, C represents for logistic regression (LR) internal validation ROC curve, D represents for LR internal validation calibration curve, E represents for decision tree (DT) internal validation ROC curve, F represents for DT internal validation calibration curve, G represents for naive Bayes (NB) internal validation ROC curve, H represents for NB internal validation calibration curve, I represents for ROC curve diagram of artificial neural network (ANN) internal verification, J represents for ANN internal verification calibration curve, K represents for K-Nearest (KNN) internal verification ROC curve, L represents for KNN internal verification ROC curve, M represents for eXtreme gradient boosting (XGBoost) internal verification ROC curve, N represents for XGBoost internal verification calibration curve, O represents gradient boosting (GraBoost) internal verification ROC curve for gradient lifting, P represents GraBoost internal verification calibration curve for gradient lifting, Q represents gradient boosting with categorical features support (CatBoost) internal verification ROC curve for gradient lifting that supports classification features, and R represents CatBoost internal verification calibration curve for gradient lifting that supports classification features. S represents for light gradient boosting machine (LGBM) internal verification ROC curve of lightweight gradient elevator, and T represents for LGBM internal verification calibration curve of lightweight gradient elevator.

**Supplemental Figure 3 ROC curves and calibration curves in external validation of different machine learning models**

Abbreviation: A represents for random forest (RF) external validation ROC curve, B represents for RF external validation calibration curve, C represents for logistic regression (LR) external validation ROC curve, D represents for LR external validation calibration curve, E represents for decision tree (DT) external validation ROC curve, F represents for DT external validation calibration curve, G represents for naive Bayes (NB) external validation ROC curve, H represents for NB external validation calibration curve, I represents for ROC curve diagram of artificial neural network (ANN) external verification, J represents for ANN external verification calibration curve, K represents for K-Nearest (KNN) external verification ROC curve, L represents for KNN external verification ROC curve, M represents for eXtreme gradient boosting (XGBoost) external verification ROC curve, N represents for XGBoost external verification calibration curve, O represents gradient boosting (GraBoost) external verification ROC curve for gradient lifting, P represents GraBoost external verification calibration curve for gradient lifting, Q represents gradient boosting with categorical features support (CatBoost) external verification ROC curve for gradient lifting that supports classification features, and R represents CatBoost external verification calibration curve for gradient lifting that supports classification features. S represents for light gradient boosting machine (LGBM) external verification ROC curve of lightweight gradient elevator, and T represents for LGBM external verification calibration curve of lightweight gradient elevator.

**Supplemental Figure 4 Brier score in internal validation of different machine learning models**

Abbreviation: RF, random forest; LR, logistic regression; DT, decision tree; NB, naive bayes; ANN, artificial neural network; KNN, K-Nearest neighbors; XGBoost, eXtreme gradient boosting; GraBoost, gradient boosting; CatBoost, gradient boosting with categorical features support; LGBM, light gradient boosting machine.

**Supplemental Figure 5. Prediction example**

**Legend of Supplemental Figure 5:** A 75-year-old patient with LEAD and diabetes, having a Barthel Index of 85, Rutherford classification R6, presenting with a wound, residing in rural/town, with severe financial burden, low PALB, normal FIB, normal APTT, high CRP, high NEUT, without diabetic foot, and not on statin therapy. The GraBoost model predicted an amputation risk probability of 44.7%, and the Fine-Gray competing-risk model predicted a 12-month amputation cumulative incidence of 39.8%. When risk values exceed the predefined thresholds, the results are highlighted in red on the interface, indicating that the patient belongs to the high amputation risk group.

**Supplemental Figure 6 ROC curve for amputation prediction model**

**Legend of Supplemental Figure 6:** A cutoff of 0.383 implies that patients scoring above this value have a 38.3% or higher probability of amputation based on the model’s calibration. Patients with predicted probabilities ≥38.3% are classified as "high-risk" of amputation occurrence. Patients with scores <38.3% are "low-risk" of amputation occurrence.
