# Supplementary material for: Development and validation of a machine learning-based model for predicting amputation risk in patients with lower extremity arterial disease and diabetes
Source: Front Cardiovasc Med. 2026 Oct 20;13:1893875. doi: 10.3389/fcvm.2026.1893875 (PMC13727486; doi:10.3389/fcvm.2026.1893875)
Supplement: Supplementary file 2 [file Datasheet2.docx]

**Supplemental Table 1 Summary of risk factors associated with amputation in patients with lower extremity arterial disease and diabetes**

| **Riskfactor** | | **No.ofliteratures** | **Literature** |
| --- | --- | --- | --- |
| **Demographicfactor** | |  |  |
|  | Age | 6 | ^[1-6]^ |
|  | Sex | 7 | ^[2-5,7-9]^ |
|  | Smoking | 8 | ^[6,9-15]^ |
|  | Drinking | 2 | ^[9,16]^ |
|  | Residence | 4 | ^[2,17-19]^ |
|  | Nationality | 5 | ^[2,17,18,20,21]^ |
|  | Financial burden | 2 | ^[18,20]^ |
| **Medical history factors** | |  |  |
|  | Hypertension | 5 | ^[11-13,15,22]^ |
|  | Hyperlipidemia | 2 | ^[3,6]^ |
|  | Diabetic Foot | 1 | ^[18]^ |
|  | Coronary Heart Disease (CHD) | 1 | ^[23]^ |
|  | Chronic Kidney Disease (CKD) | 5 | ^[3,4,6,24,25]^ |
|  | Dialysis-dependent Renal Failure (DRF) | 4 | ^[4,5,13,26]^ |
|  | Chronic Obstructive Pulmonary Disease (COPD) | 1 | ^[9]^ |
|  | Diabetic Peripheral Neuropathy (DPN) | 3 | ^[4,5,13]^ |
|  | Heart Failure (HF) | 2 | ^[23,25]^ |
|  | Myocardial Infarction (MI) | 2 | ^[23,25]^ |
|  | Percutaneous Coronary Intervention (PCI) | 2 | ^[23,25]^ |
|  | Charlson Comorbidity Index (CCI) | 1 | ^[1]^ |
|  | Diabetes type | 1 | ^[27]^ |
|  | Duration of diabetes | 4 | ^[2,4,24,28]^ |
| **Biomarkers and laboratory measurements** | |  |  |
|  | Ankle-brachial index (ABI) | 1 | ^[6,29]^ |
|  | BMI classification | 1 | ^[30]^ |
|  | Barthel Index | 3 | ^[8,31,32]^ |
|  | Hemoglobin A1c (HbA1c) | 11 | ^[3,4,6,13,18,22,27,33-36]^ |
|  | Triglycerides (TG)  Total Cholesterol (TC)  High-Density Lipoprotein (HDL)  Low-Density Lipoprotein (LDL)  Apolipoprotein A1 (ApoA1)  Apolipoprotein B (ApoB) | 8 | ^[4,6,12,15,16,28,30,37]^ |
|  | Prothrombin Time (PT)  International Normalized Ratio (INR)  Fibrinogen (FIB)  Thrombin Time (TT)  Activated Partial Thromboplastin Time (APTT)  D-Dimer | 5 | ^[5,6,16,38-40]^ |
|  | C-Reactive Protein (CRP) | 5 | ^[10,26,40]^ |
|  | Random Blood Glucose (RBG)  Fasting Blood Glucose (FBG) | 2 | ^[28,30]^ |
|  | Homocysteine (Hcy) | 2 | ^[12,41]^ |
|  | White Blood Cell Count (WBC) | 1 | ^[9]^ |
|  | Procalcitonin (PCT) | 1 | ^[42]^ |
|  | Prealbumin (PALB) | 1 | ^[40]^ |
|  | Monocyte Count (MONO) | 2 | ^[25,43]^ |
|  | Neutrophil Count (NEUT) | 4 | ^[12,13,25,40]^ |
|  | Lymphocyte Count (LYM) | 4 | ^[12,13,25,40]^ |
|  | Monocyte to HDL Ratio (MHR) | 1 | ^[44]^ |
|  | Chitinase-3-like protein 1 (CHI3L1) | 1 | ^[45]^ |
|  | Heat Shock Protein 60 (HSP60) | 1 | ^[45]^ |
|  | Cystatin C (CysC) | 1 | ^[46]^ |
|  | Ischemia Modified Albumin (IMA) | 1 | ^[46]^ |
|  | Lipoprotein-Associated Phospholipase A2 (LP-PLA2) | 1 | ^[46]^ |
|  | Uric Acid (UA) | 1 | ^[47]^ |
|  | High Mobility Group Box 1 (HMGB1) | 1 | ^[47,48]^ |
|  | Uric Acid Transporters (UAT) | 1 | ^[47]^ |
|  | Vascular Cell Adhesion Molecule-1 (VCAM-1) | 1 | ^[49]^ |
|  | Sortilin | 1 | ^[50]^ |
|  | Total Bilirubin (TB) | 1 | ^[36]^ |
| **Medication factors** | |  |  |
|  | Antiplatelet | 4 | ^[3,24,51-53]^ |
|  | Rivaroxaban | 1 | ^[9]^ |
|  | Statin | 4 | ^[3,11,24,54]^ |
|  | Cilostazol | 2 | ^[53,55]^ |
|  | Proprotein Convertase Subtilisin/Kexin type 9 inhibitors (PCSK9) | 1 | ^[3]^ |
|  | Sodium-Glucose Cotransporter-2 Inhibitors (SGLT2is) | 4 | ^[56-59]^ |
|  | Dipeptidyl Peptidase-4 Inhibitors (DPP-4is) | 2 | ^[59,60]^ |
|  | Semaglutide | 1 | ^[61]^ |
|  | Autologous cell therapy | 2 | ^[10,62]^ |
|  | Warfarin | 2 | ^[52,55]^ |
|  | Tirzepatide | 1 | ^[63]^ |
| **Other factors** | |  |  |
|  | Rutherford classification | 2 | ^[9,64]^ |
|  | Endovascular Revascularization (EVR) Number | 5 | ^[8,9,24,62,65]^ |
|  | Angiographic evaluation | 2 | ^[18,66]^ |
|  | Wound | 4 | ^[24,25,65,67]^ |
|  | Wagner classification | 1 | ^[16]^ |

**Supplemental Table 2 Variable processing workflow: clinical accessibility assessment, missingness, imputation strategy, and final inclusion for feature selection**

| **Risk factor** | **Clinically inaccessible** | **Missingness (%)** | **Handling strategy** | **Entered feature selection** |
| --- | --- | --- | --- | --- |
| Age | Yes | 0.0 | / | Yes |
| Sex | Yes | 0.0 | / | Yes |
| Smoking | Yes | 0.0 | / | Yes |
| Drinking | Yes | 27.8 | Removed | No |
| Residence | Yes | 0.0 | / | Yes |
| Nationality | Yes | 0.0 | / | Yes |
| Financial burden | Yes | 0.0 | / | Yes |
| Hypertension | Yes | 0.0 | / | Yes |
| Hyperlipidemia | Yes | 0.0 | / | Yes |
| Diabetic Foot | Yes | 0.0 | / | Yes |
| Coronary Heart Disease (CHD) | Yes | 0.0 | / | Yes |
| Chronic Kidney Disease (CKD) | Yes | 0.0 | / | Yes |
| Dialysis-dependent Renal Failure (DRF) | Yes | 0.0 | / | Yes |
| Chronic Obstructive Pulmonary Disease (COPD) | Yes | 0.0 | / | Yes |
| Diabetic Peripheral Neuropathy (DPN) | Yes | 0.0 | / | Yes |
| Heart Failure (HF) | Yes | 0.0 | / | Yes |
| Myocardial Infarction (MI) | Yes | 0.0 | / | Yes |
| Percutaneous Coronary Intervention (PCI) | Yes | 0.0 | / | Yes |
| Charlson Comorbidity Index (CCI) | Yes | 0.0 | / | Yes |
| Diabetes type | Yes | 25.7 | Removed | No |
| Duration of diabetes | Yes | 26.3 | Removed | No |
| Ankle-brachial index (ABI) | Yes | 45.8 | Removed | No |
| BMI classification | Yes | 0.0 | / | Yes |
| Barthel Index | Yes | 0.0 | / | Yes |
| Hemoglobin A1c (HbA1c) | Yes | 3.1 | Multiple imputation | Yes |
| Triglycerides (TG) | Yes | 0.0 | / | Yes |
| Total Cholesterol (TC) | Yes | 0.0 | / | Yes |
| High-Density Lipoprotein (HDL) | Yes | 0.0 | / | Yes |
| Low-Density Lipoprotein (LDL) | Yes | 0.0 | / | Yes |
| Apolipoprotein A1 (ApoA1) | Yes | 4.3 | Multiple imputation | Yes |
| Apolipoprotein B (ApoB) | Yes | 4.3 | Multiple imputation | Yes |
| Prothrombin Time (PT) | Yes | 0.0 | / | Yes |
| International Normalized Ratio (INR) | Yes | 0.0 | / | Yes |
| Fibrinogen(FIB) | Yes | 0.0 | / | Yes |
| Thrombin Time (TT) | Yes | 25.4 | Removed | No |
| Activated Partial Thromboplastin Time (APTT) | Yes | 0.0 | / | Yes |
| D-Dimer | Yes | 25.4 | Removed | No |
| C-Reactive Protein (CRP) | Yes | 0.0 | / | Yes |
| Random Blood Glucose (RBG) | Yes | 0.0 | / | Yes |
| Fasting Blood Glucose (FBG) | Yes | 0.0 | / | Yes |
| Homocysteine (Hcy) | Yes | 0.0 | / | Yes |
| White Blood Cell Count (WBC) | Yes | 0.0 | / | Yes |
| Procalcitonin (PCT) | Yes | 0.0 | / | Yes |
| Prealbumin (PALB) | Yes | 0.0 | / | Yes |
| Monocyte Count (MONO) | Yes | 0.0 | / | Yes |
| Neutrophil Count (NEUT) | Yes | 0.0 | / | Yes |
| Lymphocyte Count (LYM) | Yes | 0.0 | / | Yes |
| Monocyte to HDL Ratio (MHR) | Yes | 0.0 | / | Yes |
| Chitinase-3-like protein 1 (CHI3L1) | No | / | / | No |
| Heat Shock Protein 60 (HSP60) | No | / | / | No |
| Cystatin C (CysC) | No | / | / | No |
| Ischemia Modified Albumin (IMA) | No | / | / | No |
| Lipoprotein-Associated Phospholipase A2 (LP-PLA2) | No | / | / | No |
| Uric Acid (UA) | No | / | / | No |
| High Mobility Group Box 1 (HMGB1) | No | / | / | No |
| Uric Acid Transporters (UAT) | No | / | / | No |
| Vascular Cell Adhesion Molecule-1 (VCAM-1) | No | / | / | No |
| Sortilin | No | / | / | No |
| Total Bilirubin (TB) | No | / | / | No |
| Antiplatelet | Yes | 0.0 | / | Yes |
| Rivaroxaban | Yes | 0.0 | / | Yes |
| Statin | Yes | 0.0 | / | Yes |
| Cilostazol | Yes | 0.0 | / | Yes |
| Proprotein Convertase Subtilisin/Kexin type 9 inhibitors (PCSK9) | No | / | / | No |
| Sodium-Glucose Cotransporter-2 Inhibitors (SGLT2is) | No | / | / | No |
| Dipeptidyl Peptidase-4 Inhibitors (DPP-4is) | No | / | / | No |
| Semaglutide | No | / | / | No |
| Autologous cell therapy | No | / | / | No |
| Warfarin | No | / | / | No |
| Tirzepatide | No | / | / | No |
| Rutherford classification | Yes | 0.0 | / | Yes |
| Endovascular Revascularization (EVR) Number | Yes | 26.0 | Removed | No |
| Angiographic evaluation | No | / | / | No |
| Wound | Yes | 0.0 | / | Yes |
| Wagner classification | Yes | 27.8 | Removed | No |

**Supplemental Table 3 Baseline characteristics of the training, internal validation, and external validation cohorts**

| **Characteristics** | **Training cohort(n=967)** | **Internal validation cohort(n=414)** | **External validation cohort(n=538)** | **χ2/F** | **p** |
| --- | --- | --- | --- | --- | --- |
| **Age (year)** | 72.9±9.2 | 73.0±9.7 | 72.1±9.9 | 2.694 | 0.068 |
| **Sex** |  |  |  | 1.695 | 0.428 |
| Male | 647(66.9) | 258(62.3) | 356(66.2) |  |  |
| Female | 320(33.1) | 156(37.7) | 182(33.8) |  |  |
| **Barthel Index** | 70.6±19.8 | 71.1±18.8 | 62.9±20.6 | 31.652 | ＜0.001 |
| **Rutherford classification** |  |  |  |  |  |
| R1 | 34(3.5) | 13(3.1) | 22(4.1) | 56.592 | ＜0.001 |
| R2 | 78(8.1) | 34(8.2) | 44(8.2) |  |  |
| R3 | 178(18.4) | 78(18.8) | 61(11.3) |  |  |
| R4 | 248(25.6) | 102(24.6) | 136(25.3) |  |  |
| R5 | 416(43.0) | 178(43.0) | 235(43.7) |  |  |
| R6 | 13(1.3) | 9(2.2) | 40(7.4) |  |  |
| **BMI classification** |  |  |  | 15.787 | 0.015 |
| Underweight (<18.5) | 29(3.0) | 32(7.7) | 36(6.7) |  |  |
| Normal (18.5-23.9) | 673(69.6) | 286(69.1) | 329(61.2) |  |  |
| Overweight (24.0-27.9) | 217(22.4) | 78(18.8) | 142(26.4) |  |  |
| Obesity (≥28) | 48(5.0) | 18(4.3) | 31(5.8) |  |  |
| **Nationality** |  |  |  | 9.312 | 0.010 |
| The Han nationality | 937(96.9) | 410(99.0) | 536(99.6) |  |  |
| Ethnic minorities | 30(3.1) | 4(1.0) | 2(0.4) |  |  |
| **Smoking** |  |  |  | 0.986 | 0.611 |
| No | 457(47.3) | 212(51.2) | 261(48.5) |  |  |
| Yes | 510(52.7) | 202(48.8) | 277(51.5) |  |  |
| **Residence** |  |  |  | 80.655 | ＜0.001 |
| Rural or town | 451(46.6) | 200(48.3) | 376(69.9) |  |  |
| City | 516(53.4) | 214(51.7) | 162(30.1) |  |  |
| **Financial burden** |  |  |  | 10.237 | 0.015 |
| None | 6(0.6) | 5(1.2) | 10(1.9) |  |  |
| Mild | 228(23.6) | 102(24.9) | 142(26.4) |  |  |
| Moderate | 594(61.4) | 254(61.4) | 298(55.4) |  |  |
| Severe | 139(14.4) | 52(12.6) | 88(16.4) |  |  |
| **Wound** |  |  |  | 8.557 | 0.014 |
| No | 515(53.3) | 219(52.9) | 246(45.7) |  |  |
| Yes | 452(46.7) | 195(47.1) | 292(54.3) |  |  |
| **TG** |  |  |  | 24.102 | ＜0.001 |
| Normal | 630(65.1) | 255(61.6) | 280(52.0) |  |  |
| High | 337(34.9) | 159(38.4) | 258(48.0) |  |  |
| **TC** |  |  |  | 2.369 | 0.668 |
| Low | 26(2.7) | 17(4.1) | 12(2.2) |  |  |
| Normal | 672(69.5) | 289(69.8) | 383(71.2) |  |  |
| High | 269(27.8) | 108(26.1) | 143(26.6) |  |  |
| **HDL** |  |  |  | 25.464 | ＜0.001 |
| Low | 428(44.3) | 178(43.0) | 204(37.9) |  |  |
| Normal | 493(51.0) | 214(51.7) | 326(60.6) |  |  |
| High | 46(4.8) | 22(5.3) | 8(1.5) |  |  |
| **ApoA1** |  |  |  | 10.597 | 0.031 |
| Low | 71(7.3) | 38(9.2) | 25(4.6) |  |  |
| Normal | 450(46.5) | 178(43.0) | 227(42.2) |  |  |
| High | 446(46.1) | 198(47.8) | 286(53.2) |  |  |
| **LDL** |  |  |  | 55.563 | ＜0.001 |
| Low | 18(1.9) | 12(2.9) | 47(8.7) |  |  |
| Normal | 756(78.2) | 313(75.6) | 424(78.8) |  |  |
| High | 193(20.0) | 89(21.5) | 67(12.5) |  |  |
| **ApoB** |  |  |  | 6.111 | 0.191 |
| Low | 227(23.5) | 101(24.4) | 106(19.7) |  |  |
| Normal | 643(66.5) | 270(65.2) | 367(68.2) |  |  |
| High | 97(10.0) | 43(10.4) | 65(12.1) |  |  |
| **PT** |  |  |  | 9.184 | 0.057 |
| Low | 36(3.7) | 6(1.4) | 14(2.6) |  |  |
| Normal | 707(73.1) | 320(77.3) | 376(69.9) |  |  |
| High | 224(23.2) | 88(21.3) | 148(27.5) |  |  |
| **INR** |  |  |  | 2.696 | 0.260 |
| Normal | 921(95.2) | 395(95.4) | 503(93.5) |  |  |
| High | 46(4.8) | 19(4.6) | 35(6.5) |  |  |
| **FIB** |  |  |  | 10.156 | 0.038 |
| Low | 27(2.8) | 10(2.4) | 4(0.7) |  |  |
| Normal | 345(35.7) | 150(36.2) | 177(32.9) |  |  |
| High | 595(61.5) | 254(61.4) | 357(66.4) |  |  |
| **APTT** |  |  |  | 9.891 | 0.042 |
| Low | 121(12.5) | 42(10.1) | 81(15.1) |  |  |
| Normal | 683(70.6) | 292(70.5) | 387(71.9) |  |  |
| High | 163(16.9) | 80(19.3) | 70(13.0) |  |  |
| **CRP** |  |  |  | 91.506 | ＜0.001 |
| Low | 32(3.3) | 15(3.6) | 68(12.6) |  |  |
| Normal | 496(51.3) | 220(53.1) | 320(59.5) |  |  |
| High | 439(45.4) | 179(43.2) | 150(27.9) |  |  |
| **RBG** |  |  |  | 1.091 | 0.129 |
| Normal | 246(25.4) | 90(21.7) | 155(28.8) |  |  |
| High | 721(74.6) | 324(78.3) | 383(71.2) |  |  |
| **HbA1c** |  |  |  | 1.990 | 0.370 |
| Normal | 74(7.7) | 22(5.3) | 28(5.2) |  |  |
| High | 893(92.3) | 392(94.7) | 510(94.8) |  |  |
| **Hcy** |  |  |  | 4.128 | 0.127 |
| Normal | 859(88.8) | 366(88.4) | 465(86.4) |  |  |
| High | 108(11.2) | 48(11.6) | 73(13.6) |  |  |
| **WBC** |  |  |  | 37.754 | ＜0.001 |
| Normal | 732(75.7) | 323(78.0) | 336(62.5) |  |  |
| High | 235(24.3) | 91(22.0) | 202(37.5) |  |  |
| **PCT** |  |  |  | 74.155 | ＜0.001 |
| Normal | 819(84.7) | 365(88.2) | 370(68.8) |  |  |
| High | 148(15.3) | 49(11.8) | 168(31.2) |  |  |
| **FBG** |  |  |  | 71.086 | ＜0.001 |
| Low | 21(2.2) | 15(3.6) | 52(9.7) |  |  |
| Normal | 240(24.8) | 97(23.5) | 182(33.8) |  |  |
| High | 706(73.0) | 302(72.9) | 304(56.5) |  |  |
| **PALB** |  |  |  | 3.531 | 0.171 |
| Low | 391(40.4) | 188(45.4) | 241(44.8) |  |  |
| Normal | 576(59.6) | 226(54.6) | 297(55.2) |  |  |
| **MONO** |  |  |  | 3.897 | 0.142 |
| Normal | 626(64.7) | 264(63.8) | 355(66.0) |  |  |
| High | 341(35.3) | 150(36.2) | 183(34.0) |  |  |
| **NEUT** |  |  |  | 41.693 | ＜0.001 |
| Normal | 652(67.4) | 271(65.5) | 277(51.5) |  |  |
| High | 315(32.6) | 143(34.5) | 261(48.5) |  |  |
| **LYM** |  |  |  | 1.139 | 0.888 |
| Low | 268(27.7) | 123(29.7) | 143(26.6) |  |  |
| Normal | 684(70.7) | 282(68.1) | 383(71.2) |  |  |
| High | 15(1.6) | 9(2.2) | 12(2.2) |  |  |
| **MHR** |  |  |  | 0.520 | 0.771 |
| Normal | 347(35.9) | 155(37.4) | 199(37.0) |  |  |
| High | 620(64.1) | 259(62.6) | 339(63.0) |  |  |
| **Hypertension** |  |  |  | 9.272 | 0.010 |
| No | 200(20.7) | 87(21.0) | 146(27.1) |  |  |
| Yes | 767(79.3) | 327(79.0) | 392(72.9) |  |  |
| **Hyperlipidemia** |  |  |  | 39.656 | ＜0.001 |
| No | 770(79.6) | 330(79.7) | 491(91.3) |  |  |
| Yes | 197(20.4) | 84(20.3) | 47(8.7) |  |  |
| CHD |  |  |  | 5.113 | 0.078 |
| No | 680(70.3) | 292(70.5) | 405(75.3) |  |  |
| Yes | 287(29.7) | 122(29.5) | 133(24.7) |  |  |
| **Diabetic Foot** |  |  |  | 31.772 | ＜0.001 |
| No | 662(68.5) | 291(70.3) | 440(81.8) |  |  |
| Yes | 305(31.5) | 123(29.7) | 98(18.2) |  |  |
| **CKD** |  |  |  | 57.360 | ＜0.001 |
| No | 778(80.5) | 335(80.9) | 506(94.1) |  |  |
| Yes | 189(19.5) | 79(19.1) | 32(5.9) |  |  |
| **DRF** |  |  |  | 15.871 | ＜0.001 |
| No | 919(95.0) | 384(92.8) | 530(98.5) |  |  |
| Yes | 48(5.0) | 30(7.2) | 8(1.5) |  |  |
| **COPD** |  |  |  | 44.110 | ＜0.001 |
| No | 823(85.1) | 340(82.1) | 513(95.4) |  |  |
| Yes | 144(14.9) | 74(17.9) | 25(4.6) |  |  |
| **DPN** |  |  |  | 64.390 | ＜0.001 |
| No | 757(78.3) | 323(78.0) | 504(93.7) |  |  |
| Yes | 210(21.7) | 91(22.0) | 34(6.3) |  |  |
| **HF** |  |  |  | 0.177 | 0.915 |
| No | 959(99.2) | 409(98.8) | 534(99.3) |  |  |
| Yes | 8(0.8) | 5(1.2) | 4(0.7) |  |  |
| **MI** |  |  |  | 4.066 | 0.131 |
| No | 955(98.8) | 402(97.1) | 533(99.1) |  |  |
| Yes | 12(1.2) | 12(2.9) | 5(0.9) |  |  |
| **PCI** |  |  |  | 2.510 | 0.285 |
| No | 858(88.7) | 374(90.3) | 493(91.6) |  |  |
| Yes | 109(11.3) | 40(9.7) | 45(8.4) |  |  |
| **CCI** |  |  |  | 83.776 | ＜0.001 |
| Mild | 68(7.0) | 22(5.3) | 62(11.5) |  |  |
| Moderate | 346(35.8) | 144(34.8) | 288(53.5) |  |  |
| Severe | 553(57.2) | 248(59.9) | 188(34.9) |  |  |
| **Antiplatelet** |  |  |  | 0.922 | 0.631 |
| No | 215(22.2) | 86(20.8) | 123(22.9) |  |  |
| Yes | 752(77.8) | 328(79.2) | 415(77.1) |  |  |
| **Rivaroxaban** |  |  |  | 222.294 | ＜0.001 |
| No | 785(81.2) | 327(79.0) | 248(46.1) |  |  |
| Yes | 182(18.8) | 87(21.0) | 290(53.9) |  |  |
| **Statin** |  |  |  | 53.629 | ＜0.001 |
| No | 415(42.9) | 193(46.6) | 140(26.0) |  |  |
| Yes | 552(57.1) | 221(53.4) | 398(74.0) |  |  |
| **Cilostazol** |  |  |  | 92.080 | ＜0.001 |
| No | 785(81.2) | 337(81.4) | 528(98.1) |  |  |
| Yes | 182(18.8) | 77(18.6) | 10(1.9) |  |  |

Abbreviation: BMI: body mass index; TG: triglyceride; TC: total cholesterol; HDL: High-density lipoprotein; ApoA1: apolipoprotein A1; LDL: Low-density lipoprotein; ApoB: apolipoprotein B; PT: prothrombin time; INR: international normalized ratio; FIB: fibrinogen; APTT: activated partial thromboplastin time; CRP: C-reactive protein; RBG: random blood glucose; HbA1c: hemoglobin A1c; Hcy: homocysteine; WBC: white blood cell; PCT: procalcitonin; FBG: fasting blood glucose; PALB: prealbumin; MONO: monocyte; NEUT: neutrophil; LYM: lymphocyte; MHR: monocyte to HDL cholesterol ratio (MONO/HDL); CHD: coronary heart disease; CKD: chronic kidney disease; COPD: chronic obstructive pulmonary disease; DRF: dialysis-dependent renal failure; DPN: diabetic peripheral neuropathy; HF: heart failure; MI: myocardial infarction; PCI: percutaneous coronary intervention; CCI: charlson comorbidity index.

**Supplemental Table 4 Baseline characteristics of the overall cohort compared with patients lost to follow-up**

| **Characteristics** | **overall cohort**  **(n=1,919)** | **lost to follow-up**  **(n=597)** | **χ2/*F*** | ***p*** |
| --- | --- | --- | --- | --- |
| **Age (year)** | 72.7±9.5 | 72.9±9.3 | 23.809 | ＜0.001 |
| **Sex** |  |  | 1.597 | 0.206 |
| Male | 1261(65.7) |  |  |  |
| Female | 658(34.3) |  |  |  |
| **Barthel Index** | 68.5±20.1 | 71.7±19.1 | 11.526 | ＜0.001 |
| **Rutherford classification** |  |  | 23.488 | ＜0.001 |
| R1 | 69(3.6) | 7(1.2) |  |  |
| R2 | 156(8.1) | 42(7.0) |  |  |
| R3 | 317(16.5) | 103(17.3) |  |  |
| R4 | 486(25.3) | 191(32.0) |  |  |
| R5 | 829(43.2) | 246(41.2) |  |  |
| R6 | 62(3.2) | 8(1.3) |  |  |
| **BMI classification** |  |  | 17.901 | ＜0.001 |
| Underweight (<18.5) | 97(5.1) | 36(6.0) |  |  |
| Normal (18.5-23.9) | 1288(67.1) | 446(74.7) |  |  |
| Overweight (24.0-27.9) | 437(22.8) | 97(16.2) |  |  |
| Obesity (≥28) | 97(5.1) | 18(3.0) |  |  |
| **Nationality** |  |  | 2.813 | 0.093 |
| The Han nationality | 1883(98.1) | 579(97.0) |  |  |
| Ethnic minorities | 36(1.9) | 18(3.0) |  |  |
| **Smoking** |  |  | 0.056 | 0.812 |
| No | 930(48.5) | 286(47.9) |  |  |
| Yes | 989(51.5) | 251(42.0) |  |  |
| **Residence** |  |  | 8.897 | 0.003 |
| Rural or town | 1027(53.5) | 361(60.5) |  |  |
| City | 892(46.5) | 236(39.5) |  |  |
| **Financial burden** |  |  | 49.249 | ＜0.001 |
| None | 21(1.1) | 2(0.3) |  |  |
| Mild | 473(24.6) | 92(15.4) |  |  |
| Moderate | 1146(59.7) | 354(59.3) |  |  |
| Severe | 279(14.5) | 149(25.0) |  |  |
| **Wound** |  |  | 8.667 | 0.003 |
| No | 980(51.1) | 356(58.0) |  |  |
| Yes | 939(48.9) | 251(42.0) |  |  |
| **TG** |  |  | 0.063 | 0.802 |
| Normal | 1165(60.7) | 359(60.1) |  |  |
| High | 754(39.3) | 238(39.9) |  |  |
| **TC** |  |  | 28.119 | ＜0.001 |
| Low | 55(2.9) | 389(65.2) |  |  |
| Normal | 1344(70.0) | 208(34.8) |  |  |
| High | 520(27.1) |  |  |  |
| **HDL** |  |  | 25.468 | ＜0.001 |
| Low | 810(42.2) | 0(0.0) |  |  |
| Normal | 1033(53.8) | 248(41.5) |  |  |
| High | 76(4.0) | 349(58.5) |  |  |
| **ApoA1** |  |  | 58.338 | ＜0.001 |
| Low | 134(7.0) | 17(2.8) |  |  |
| Normal | 855(44.6) | 187(31.3) |  |  |
| High | 930(48.5) | 393(65.8) |  |  |
| **LDL** |  |  | 24.910 | ＜0.001 |
| Low | 77(4.0) | 0(0.0) |  |  |
| Normal | 1493(77.8) | 479(80.2) |  |  |
| High | 349(18.2) | 118(19.8) |  |  |
| **ApoB** |  |  | 41.325 | ＜0.001 |
| Low | 434(22.6) | 91(15.2) |  |  |
| Normal | 1280(66.7) | 388(65.0) |  |  |
| High | 205(10.7) | 118(19.8) |  |  |
| **PT** |  |  | 32.807 | ＜0.001 |
| Low | 56(2.9) | 11(1.8) |  |  |
| Normal | 1403(73.1) | 505(84.6) |  |  |
| High | 460(24.0) | 81(13.6) |  |  |
| **INR** |  |  | 0.450 | 0.502 |
| Normal | 1819(94.8) | 570(95.5) |  |  |
| High | 100(5.2) | 27(4.5) |  |  |
| **FIB** |  |  | 5.308 | 0.070 |
| Low | 41(2.1) | 6(1.0) |  |  |
| Normal | 672(35.0) | 231(38.7) |  |  |
| High | 1206(62.8) | 360(60.3) |  |  |
| **APTT** |  |  | 1.809 | 0.405 |
| Low | 244(12.7) | 87(14.6) |  |  |
| Normal | 1362(71.0) | 408(68.3) |  |  |
| High | 313(16.3) | 102(17.1) |  |  |
| **CRP** |  |  | 42.693 | ＜0.001 |
| Low | 115(6.0) | 0(0.0) |  |  |
| Normal | 1036(54.0) | 374(62.6) |  |  |
| High | 768(40.0) | 223(37.4) |  |  |
| **RBG** |  |  | 138.158 | ＜0.001 |
| Normal | 491(25.6) | 298(49.9) |  |  |
| High | 1428(74.4) | 299(50.1) |  |  |
| **HbA1c** |  |  | 601.661 | ＜0.001 |
| Normal | 124(6.5) | 294(49.2) |  |  |
| High | 1795(93.5) | 303(50.8) |  |  |
| **Hcy** |  |  | 2.955 | 0.086 |
| Normal | 1690(88.1) | 541(90.6) |  |  |
| High | 229(11.9) | 56(9.4) |  |  |
| **WBC** |  |  | 3.240 | 0.072 |
| Normal | 1391(72.5) | 455(76.2) |  |  |
| High | 528(27.5) | 142(23.8) |  |  |
| **PCT** |  |  | 29.269 | ＜0.001 |
| Normal | 1554(81.0) | 540(90.5) |  |  |
| High | 365(19.0) | 57(9.5) |  |  |
| **FBG** |  |  | 34.535 | ＜0.001 |
| Low | 88(4.6) | 221(37.0) |  |  |
| Normal | 519(27.0) | 376(63.0) |  |  |
| High | 1312(68.4) |  |  |  |
| **PALB** |  |  | 0.619 | 0.432 |
| Low | 820(42.7) | 266(44.6) |  |  |
| Normal | 1099(57.3) | 331(55.4) |  |  |
| **MONO** |  |  | 4.128 | 0.042 |
| Normal | 1245(64.9) | 360(60.3) |  |  |
| High | 674(35.1) | 237(39.7) |  |  |
| **NEUT** |  |  | 14.919 | ＜0.001 |
| Normal | 1200(62.5) | 425(71.2) |  |  |
| High | 719(37.5) | 172(28.8) |  |  |
| **LYM** |  |  | 4.315 | 0.116 |
| Low | 53427.8) | 182(30.5) |  |  |
| Normal | 1349(70.3) | 410(68.7) |  |  |
| High | 36(1.9) | 5(0.8) |  |  |
| **MHR** |  |  | 7.794 | 0.005 |
| Normal | 701(36.5) | 256(42.9) |  |  |
| High | 1218(63.5) | 341(57.1) |  |  |
| **Hypertension** |  |  | 3.537 | 0.060 |
| No | 433(22.6) | 157(26.3) |  |  |
| Yes | 1486(77.4) | 440(73.7) |  |  |
| **Hyperlipidemia** |  |  | 2.231 | 0.135 |
| No | 1591(82.9) | 479(80.2) |  |  |
| Yes | 328(17.1) | 118(19.8) |  |  |
| **CHD** |  |  | 3.904 | 0.048 |
| No | 1377(71.8) | 453(75.9) |  |  |
| Yes | 542(28.2) | 144(24.1) |  |  |
| **Diabetic Foot** |  |  | 53.909 | ＜0.001 |
| No | 1393(72.6) | 521(87.3) |  |  |
| Yes | 526(27.4) | 76(12.7) |  |  |
| **CKD** |  |  | 5.544 | 0.019 |
| No | 1619(84.4) | 527(88.3) |  |  |
| Yes | 300(15.6) | 70(11.7) |  |  |
| **DRF** |  |  | 5.446 | 0.020 |
| No | 1833(95.5) | 583(97.7) |  |  |
| Yes | 86(4.5) | 14(2.3) |  |  |
| **COPD** |  |  | 14.714 | ＜0.001 |
| No | 1676(87.3) | 484(81.1) |  |  |
| Yes | 243(12.7) | 113(18.9) |  |  |
| **DPN** |  |  | 15.487 | ＜0.001 |
| No | 1584(82.5) | 533(89.3) |  |  |
| Yes | 335(17.5) | 64(10.7) |  |  |
| **HF** |  |  | 0.012 | 0.912 |
| No | 1902(99.1) | 592(99.2) |  |  |
| Yes | 17(0.9) | 5(0.8) |  |  |
| **MI** |  |  | 1.550 | 0.213 |
| No | 1890(98.5) | 592(99.2) |  |  |
| Yes | 29(1.5) | 5(0.8) |  |  |
| **PCI** |  |  | 3.503 | 0.061 |
| No | 1725(89.9) | 552(92.5) |  |  |
| Yes | 194(10.1) | 45(7.5) |  |  |
| **CCI** |  |  | 17.879 | ＜0.001 |
| Mild | 152(7.9) | 2(0.3) |  |  |
| Moderate | 778(40.5) | 48(8.0) |  |  |
| Severe | 989(51.5) | 286(47.9) |  |  |
| **Antiplatelet** |  |  | 1.448 | 0.229 |
| No | 424(22.1) | 146(24.5) |  |  |
| Yes | 1495(77.9) | 451(75.5) |  |  |
| **Rivaroxaban** |  |  | 39.201 | ＜0.001 |
| No | 1360(70.9) | 500(83.8) |  |  |
| Yes | 559(29.1) | 97(16.2) |  |  |
| **Statin** |  |  | 80.140 | ＜0.001 |
| No | 748(39.0) | 357(59.8) |  |  |
| Yes | 1171(61.0) | 240(40.2) |  |  |
| **Cilostazol** |  |  | 7.971 | 0.005 |
| No | 1650(86.0) | 485(81.2) |  |  |
| Yes | 269(14.0) | 112(18.8) |  |  |

Abbreviation: BMI: body mass index; TG: triglyceride; TC: total cholesterol; HDL: High-density lipoprotein; ApoA1: apolipoprotein A1; LDL: Low-density lipoprotein; ApoB: apolipoprotein B; PT: prothrombin time; INR: international normalized ratio; FIB: fibrinogen; APTT: activated partial thromboplastin time; CRP: C-reactive protein; RBG: random blood glucose; HbA1c: hemoglobin A1c; Hcy: homocysteine; WBC: white blood cell; PCT: procalcitonin; FBG: fasting blood glucose; PALB: prealbumin; MONO: monocyte; NEUT: neutrophil; LYM: lymphocyte; MHR: monocyte to HDL cholesterol ratio(MONO/HDL); CHD: coronary heart disease; CKD: chronic kidney disease; COPD: chronic obstructive pulmonary disease; DRF: dialysis-dependent renal failure; DPN: diabetic peripheral neuropathy; HF: heart failure; MI: myocardial infarction; PCI: percutaneous coronary intervention; CCI: charlson comorbidity index.

**Supplemental Table 5 Summary of risk factors associated with amputation in patients with lower extremity arterial disease and diabetes**

|  | Predictive varibles | values | Valuable explanation |
| --- | --- | --- | --- |
| 1. | Age | Continuous (years) | Age refers to the baseline age recorded at hospital admission. This baseline age was recorded as a continuous variable in years. |
| 2. | Barthel Index | Continuous (0-100) | The Barthel Index is a standardized tool used to measure a person's level of independence in ten essential Activities of Daily Living. The total score ranges from 0 to 100, with a higher score indicating greater functional independence.  It assesses the following ten activities, each with a specific scoring system based on the amount of assistance required:  Feeding is scored out of 10, evaluating the ability to use utensils and swallow. A patient can be independent, need help with preparation, or be fully dependent.  Bathing is a simpler item scored out of 5, distinguishing between those who can wash themselves independently and those who need assistance.  Personal Hygiene, also scored out of 5, covers activities like washing one's face and brushing teeth, rating independence versus the need for help.  Dressing is scored out of 10, assessing the ability to put on and fasten clothes and shoes, with points given for partial or full independence.  Bowel Control is scored out of 10, based on the patient's ability to control their bowels without accidents, with points deducted for occasional or full incontinence.  Bladder Control is similarly scored out of 10, rating continence or the frequency of urinary accidents.  Toileting, scored out of 10, assesses the ability to get on and off the toilet and manage personal hygiene independently.  Transfer from Bed to Chair is a key item scored out of 15. It evaluates the ability to move safely, with points given for independence, needing minor help from one person, requiring major help from two people, or being unable to transfer.  Mobility on Level Surfaces is scored out of 15. It measures the ability to walk over 50 meters, with options for independent walking, walking with help, independent wheelchair use, or being immobile.  Stairs, scored out of 10, assesses the ability to go up and down a flight of stairs, rating full independence, the need for supervision or help, or an inability to manage stairs^[68]^. |
| 3. | Rutherford classification | 1=“R1”，  2=“R2”,  3=“R3”，  4=“R4”,  5=“R5”，  6=“R6”, | R1:Mild claudication  R2:Moderate claudication  R3:Severe claudication  R4:Ischemic rest pain  R5:Minor tissue loss—nonhealing ulcer, focal gangrene with diffuse pedal ischemia  R6:Major tissue loss-xtending above transmetatarsal level, functional foot no longer salvageable |
| 4. | Wound | 0 = “No”, 1 = “Yes” | 1 represents patients had ulcer and other wound in low extremity. |
| 5. | Sex | 1 = “Male”,  2 = “Female” | 1 represents male patient, 2 represents female patient. |
| 6. | Nationality | 1 = “The Han nationality”,  2 = “Ethnic minorities” | 1 represents the Han nationality, 2 represents ethnic minorities. |
| 7. | Smoking | 0 = “No”, 1 = “Yes” | Current smoking was defined as active tobacco use (≥1 cigarette/day) within 30 days prior to baseline assessment.1 represents active smoking status at baseline. |
| 8. | Residence | 1 = “Rural or Town”, 2 = “City” | 1 represents that the patient has been living in a rural area or living in a town for a long time. 2 represents that the patient lives in a city for a long time. |
| 9. | Financial burden | 1 = “None”, 2 = “Mild”, 3 = “Moderate”, 4 = “Severe” | 1 represents that the patient has no financial burden. 2 represents that the patient has a mild financial burden. 3 represents that the patient has a moderated financial burden. 4 represents that the patient has a severe financial burden. |
| 10. | BMI classification | 1 = “<18.5”,  2 = “18.5-23.9”,  3 = “24.0-27.9”,  4 = “≥28” | BMI is short for body mass index classification. The body mass index classification was graded as the patient's condition at admission. 1 represents underweight, 2 represents normal, 3 represents overweight, 4 represents obesity. |
| 11. | TG | 2 = “Normal”,  3 = “High” | TG is short for triglyceride. 2 indicates that the triglyceride value is 0.29-1.7 mmol/L. 3 indicates that the triglyceride value > 1.7mmol/L. |
| 12. | TC | 1 = “Low”,  2 = “Normal”,  3 = “High” | TC is short for total cholesterol. 1 indicates that the total cholesterol value is < 2.8mmol/L, 2 indicates that the total cholesterol value is 2.8-5.2mmol/L, and 3 indicates that the total cholesterol value is > 5.2mmol/L. |
| 13. | HDL | 1 = “Low”,  2 = “Normal”,  3 = “High” | HDL is short for High-density lipoprotein.1 indicates a high density lipoprotein value < 1.04mmol/L, 2 indicates a high density lipoprotein value of 1.04-2.08mmol/L, and 3 indicates a high density lipoprotein value > 2.08mmol/L. |
| 14. | ApoA1 | 1 = “Low”,  2 = “Normal”,  3 = “High” | ApoA1 is short for apolipoprotein A1.1 indicates that the value of apolipoprotein A1 is <1.0g/L, 2 indicates that the value of apolipoprotein A1 is 1.0-1.6g/L, and 3 indicates that the value of apolipoprotein A1 is > 1.6g/L. |
| 15. | LDL | 1 = “Low”,  2 = “Normal”,  3 = “High” | LDL is short for Low-density lipoprotein. 1 indicates a low density lipoprotein value < 2.7mmol/L, 2 indicates a low density lipoprotein value of 2.7-3.1mmol/L, and 3 indicates a low density lipoprotein value > 3.1mmol/L. |
| 16. | ApoB | 1 = “Low”,  2 = “Normal”,  3 = “High” | ApoB is short for apolipoprotein B.1 indicates that the value of apolipoprotein B is < 0.8g/L. 2 indicates that the value of apolipoprotein B is 0.8-1.5g/L. 3 indicates that the value of apolipoprotein B is > 1.5g/L. |
| 17. | PT | 1 = “Low”,  2 = “Normal”,  3 = “High” | PT is short for prothrombin time.1 indicates that the value of prothrombin time is < 11.0s. 2 indicates that the value of prothrombin time is 11.0-15.0s. 3 indicates that the value of prothrombin time is > 15.0s. |
| 18. | INR | 2 = “Normal”,  3 = “High” | INR is short for international normalized ratio. 2 indicates that the value of international normalized ratio is 0.8-1.2. 3 indicates that the value of international normalized ratio is > 1.2. |
| 19. | FIB | 1 = “Low”,  2 = “Normal”,  3 = “High” | FIB is short for fibrinogen.1 indicates that the value of fibrinogen is < 2.0g/L. 2 indicates that the value of fibrinogen is 2.0-4.0g/L. 3 indicates that the value of fibrinogen is > 4.0 g/L. |
| 20. | APTT | 1 = “Low”,  2 = “Normal”,  3 = “High” | APTT is short for activated partial thromboplastin time.1 indicates that the value of activated partial thromboplastin time is < 24.0s. 2 indicates that the value of activated partial thromboplastin time is 24.0-43.0s. 3 indicates that the value of activated partial thromboplastin time is > 43.0s. |
| 21. | CRP | 1 = “Low”,  2 = “Normal”,  3 = “High” | CRP is short for C-reactive protein. 3 indicates that the C-reactive protein value is > 10mg/L |
| 22. | RBG | 2 = “Normal”,  3 = “High” | RBG is short for random blood glucose. 2 indicates that the value of random blood glucose is 3.9-11.1 mmol/L. 3 indicates that the value of random blood glucose is > 11.1 mmol/L. |
| 23. | HbA1c | 2 = “Normal”,  3 = “High” | HbA1c is short for hemoglobin A1c. 2 means that the HbA1c value is 4-6%, and 3 means that the HBA1c value is > 6%. |
| 24. | Hcy | 2 = “Normal”,  3 = “High” | HCY is short for homocysteine.2 indicates that the value of homocysteine is 0-20μmol/L. 3 indicates that the value of homocysteine is > 20μmol/L. |
| 25. | WBC | 2 = “Normal”,  3 = “High” | WBC is short for white blood cell. 2 indicates the value of white blood cell is 3.5-9.5$\times{10}^{9}$/L. 3 indicates the value of white blood cell is > 9.5$\times{10}^{9}$/L. |
| 26. | PCT | 2 = “Normal”,  3 = “High” | PCT is short for procalcitonin. 2 indicates that the value of procalcitonin is 0-0.05ng/ml. 3 indicates that the value of procalcitonin is > 0.05ng/ml. |
| 27. | FBG | 1 = “Low”,  2 = “Normal”,  3 = “High” | FBG is short for fasting blood glucose.1 indicates the value of fasting blood glucose is <3.9 mmol/L. 2 indicates the value of fasting blood glucose is 3.9-11.1 mmol/L. 3 indicates the value of fasting blood glucose is > 11.1 mmol/L. |
| 28. | PALB | 2 = “Normal”,  3 = “High” | PALB is short for prealbumin. 2 indicates the value of prealbumin is 200-400mg/L. 3 indicates the value of prealbumin is > 400mg/L. |
| 29. | MONO | 2 = “Normal”,  3 = “High” | MONO is short for monocyte. 2 indicates the value of monocyte is 0.1-0.6$\times{10}^{9}$/L, and 3 indicates the value of monocyte is > 0.6$\times{10}^{9}$/L. |
| 30. | NEUT | 2 = “Normal”,  3 = “High” | NEUT is short for neutrophil. 2 indicates a neutrophil value of 1.8-6.3$\times{10}^{9}$/L, and 3 indicates a neutrophil value > 6.3$\times{10}^{9}$/L. |
| 31. | LYM | 1 = “Low”,  2 = “Normal”,  3 = “High” | LYM is short for lymphocyte.1 indicates the value of lymphocyte is < 1.1$\times{10}^{9}$/L, 2 indicates the value of lymphocyte is 1.1-3.2$\times{10}^{9}$/L, 3 indicates the value of lymphocyte is >3.2$\times{10}^{9}$/L. |
| 32. | MHR | 2 = “Normal”, 3 = “High” | MHR is short for monocyte to HDL cholesterol ratio(MONO/HDL).2 indicates the value of MHR is < 0.388, 3 indicates the value of MHR is ≥0.388. |
| 33. | Hypertension | 0 = “No”, 1 = “Yes” | 1 represents the patient’s diagnosis included hypertension on baseline. |
| 34. | Diabetic foot | 0 = “No”, 1 = “Yes” | 1 represents the patient’s diagnosis included diabetic foot on baseline. |
| 35. | Hyperlipidemia | 0 = “No”, 1 = “Yes” | 1 represents the patient’s diagnosis included hyperlipidemia on baseline. |
| 36. | CHD | 0 = “No”, 1 = “Yes” | 1 represents the patient’s diagnosis included coronary heart disease on baseline. |
| 37. | CKD | 0 = “No”, 1 = “Yes” | CKD is short for chronic kidney disease. 1 represents the patient’s diagnosis included chronic kidney disease on baseline. |
| 38. | COPD | 0 = “No”, 1 = “Yes” | COPD is short for chronic obstructive pulmonary disease. 1 represents the patient’s diagnosis included chronic obstructive pulmonary disease on baseline. |
| 39. | DRF | 0 = “No”, 1 = “Yes” | DRF is short for dialysis-dependent renal failure. 1 represents the patient’s diagnosis included dialysis-dependent renal failure on baseline. |
| 40. | DPN | 0 = “No”, 1 = “Yes” | DPN is short for diabetic peripheral neuropathy. 1 represents the patient’s diagnosis includeddiabetic peripheral neuropathy on baseline. |
| 41. | HF | 0 = “No”, 1 = “Yes” | HF is short for heart failure. 1 represents the patient’s diagnosis included heart failure on baseline. |
| 42. | MI | 0 = “No”, 1 = “Yes” | MI is short for myocardial infarction. 1 represents the patient’s diagnosis included myocardial infarction on baseline. |
| 43. | PCI | 0 = “No”, 1 = “Yes” | PCI is short for percutaneous coronary intervention. 1 represents patients with a history of percutaneous coronary intervention at baseline. |
| 44. | CCI | 1 = “Mild”, 2 = “Moderate”, 3 = “Severe” | CCI is short for charlson comorbidity index. 1 represents that the patient has a mild comorbidity burden. 2 represents that the patient has a moderated comorbidity burden. 3 represents that the patient has a severe comorbidity burden ^[69]^. |
| 45. | Antiplatelet | 0 = “No”, 1 = “Yes” | 1 represents patients accepted antiplatelet therapy on baseline. Antiplatelet drugs such as aspirin, clopidogrel, etc. |
| 46. | Rivaroxaban | 0 = “No”, 1 = “Yes” | 1 represents patients accepted rivaroxaban therapy on baseline. |
| 47. | Statin | 0 = “No”, 1 = “Yes” | 1 represents the patients accepted statin therapy on baseline. Statin drugs such as atorvastatin, rosuvastatin and so on. |
| 48. | Cilostazol | 0 = “No”, 1 = “Yes” | 1 represents patients accepted cilostazol therapy on baseline. |

**Supplemental Table 6 Variable Selection of Amputation Prediction Model (n=967)**

| **Variables** | **LASSO** | **Random forest** | **Boruta** | **Variables in optimal Amputation prediction model** |
| --- | --- | --- | --- | --- |
| Age | ✓ | ✓ | ✓ | ✓ |
| Barthel Index |  | ✓ | ✓ | ✓ |
| Rutherford classification | ✓ | ✓ | ✓ | ✓ |
| Wound | ✓ | ✓ | ✓ | ✓ |
| Sex |  |  |  |  |
| Nationality |  |  |  |  |
| Smoking |  |  |  |  |
| Residence | ✓ |  | ✓ | ✓ |
| Financial burden | ✓ | ✓ | ✓ | ✓ |
| BMI classification |  | ✓ | ✓ | ✓ |
| TG |  |  |  |  |
| TC |  |  |  |  |
| HDL | ✓ |  |  |  |
| ApoA1 |  | ✓ |  |  |
| LDL |  |  |  |  |
| ApoB |  | ✓ |  |  |
| PT |  |  |  |  |
| INR |  |  |  |  |
| FIB | ✓ | ✓ | ✓ | ✓ |
| APTT | ✓ | ✓ | ✓ | ✓ |
| CRP | ✓ |  | ✓ | ✓ |
| RBG | ✓ |  |  |  |
| HbA1c |  |  |  |  |
| Hcy | ✓ |  |  |  |
| WBC |  |  | ✓ |  |
| PCT |  |  |  |  |
| FBG |  |  |  |  |
| PALB | ✓ |  | ✓ | ✓ |
| MONO |  |  |  |  |
| NEUT | ✓ | ✓ | ✓ | ✓ |
| LYM |  | ✓ |  |  |
| MHR |  |  | ✓ |  |
| Hypertension |  |  |  |  |
| Diabetic foot | ✓ | ✓ | ✓ | ✓ |
| Hyperlipidemia |  |  |  |  |
| CHD |  |  |  |  |
| CKD |  |  |  |  |
| COPD |  |  |  |  |
| DRF |  |  |  |  |
| DPN |  |  |  |  |
| HF | ✓ |  |  |  |
| MI |  |  |  |  |
| PCI |  |  |  |  |
| CCI |  | ✓ |  |  |
| Antiplatelet |  |  |  |  |
| Rivaroxaban |  |  |  |  |
| Statin | ✓ | ✓ |  | ✓ |
| Cilostazol |  |  |  |  |
| Total | 16 | 15 | 15 | 14 |

Abbreviation: ✓represents variables included in the variable selection. BMI: body mass index; TG: triglyceride; TC: total cholesterol; HDL: High-density lipoprotein; ApoA1: apolipoprotein A1; LDL: Low-density lipoprotein; ApoB: apolipoprotein B; PT: prothrombin time; INR: international normalized ratio; FIB: fibrinogen; APTT: activated partial thromboplastin time; CRP: C-reactive protein; RBG: random blood glucose; HbA1c: hemoglobin A1c; Hcy: homocysteine; WBC: white blood cell; PCT: procalcitonin; FBG: fasting blood glucose; PALB: prealbumin; MONO: monocyte; NEUT: neutrophil; LYM: lymphocyte; MHR: monocyte to HDL cholesterol ratio (MONO/HDL); CHD: coronary heart disease; CKD: chronic kidney disease; COPD: chronic obstructive pulmonary disease; DRF: dialysis-dependent renal failure; DPN: diabetic peripheral neuropathy; HF: heart failure; MI: myocardial infarction; PCI: percutaneous coronary intervention; CCI: charlson comorbidity index.

**Supplemental Table 7 DeLong's test comparing GraBoost with nine machine learning models**

|  | GraBoost  (Internal validation) | | GraBoost  (External validation) | |
| --- | --- | --- | --- | --- |
|  | Z-score | *p*-value | Z-score | *p*-value |
| RF | 4.600 | ＜0.001 | 1.763 | 0.078 |
| LR | 0.567 | 0.571 | 3.812 | ＜0.001 |
| DT | 11.469 | ＜0.001 | 8.963 | ＜0.001 |
| NB | 2.691 | 0.007 | 4.051 | ＜0.001 |
| ANN | 3.684 | ＜0.001 | 6.106 | ＜0.001 |
| KNN | 13.489 | ＜0.001 | 17.177 | ＜0.001 |
| XGBoost | 6.494 | ＜0.001 | 4.290 | ＜0.001 |
| CatBoost | 2.325 | 0.020 | 3.139 | 0.002 |
| LGBM | 6.236 | ＜0.001 | 3.141 | 0.002 |

Abbreviation: RF, random forest. LR, logistic regression. DT, decision tree. NB, naive bayes. ANN, artificial neural network. KNN, K-Nearest neighbors. XGBoost, eXtreme gradient boosting. GraBoost, gradient boosting. CatBoost, gradient boosting with categorical features support. LGBM, light gradient boosting machine.

**References**

[1] Örneholm H, Mevik J, Wenger D. Above-ankle Reamputation and Mortality following Transmetatarsal Amputation in Diabetic and Nondiabetic Peripheral Artery Disease[J]. J Foot Ankle Surg, 2024, 63(5): 584-592.

[2] Stoberock K, Kaschwich M, Nicolay S S, Mahmoud N, Heidemann F, Rieß H C, et al. The interrelationship between diabetes mellitus and peripheral arterial disease[J]. Vasa, 2021, 50(5): 323-330.

[3] Schmitt V H, Hobohm L, Vosseler M, Brochhausen C, Münzel T, Espinola-Klein C, et al. Temporal trends in patients with peripheral artery disease influenced by diabetes mellitus in Germany[J]. J Diabetes, 2022, 14(10): 670-684.

[4] Cardoso C R L, Melo J V, Santos T R M, Leite N C, Salles G F. Traditional and non-traditional risk factors for peripheral artery disease development/progression in patients with type 2 diabetes: the Rio de Janeiro type 2 diabetes cohort study[J]. Cardiovasc Diabetol, 2021, 20(1): 54.

[5] Jiahui M, Jingsi A, Huan L. Relationship between fibrinogen, PR interval and lower extremity arteriosclerosis obliterans in elderly patients with type2 diabetes mellitus(In Chinese)[J]. Jiangsu Med J, 2024, 50(05): 483-487.

[6] Jiangtao L, Xu Z, Qiang C, Yuan2 G, Kaifeng H. Analysis of Prognosis and Influencing Factors of Restenosis after Endovascular Intervention in Diabetes Patients with Lower Extremity Arteriosclerosis Obliterans(In Chinese)[J]. Progress in Modern Biomedicine, 2024, 24(16): 3118-3122.

[7] Humphries M D, Brunson A, Hedayati N, Romano P, Melnkow J. Amputation Risk in Patients with Diabetes Mellitus and Peripheral Artery Disease Using Statewide Data[J]. Ann Vasc Surg, 2016, 30: 123-31.

[8] Birmpili P, Li Q, Johal A S, Atkins E, Waton S, Chetter I, et al. Outcomes after minor lower limb amputation for peripheral arterial disease and diabetes: population-based cohort study[J]. Br J Surg, 2023, 110(8): 958-965.

[9] Czerniecki J M, Thompson M L, Littman A J, Boyko E J, Landry G J, Henderson W G, et al. Predicting reamputation risk in patients undergoing lower extremity amputation due to the complications of peripheral artery disease and/or diabetes[J]. Br J Surg, 2019, 106(8): 1026-1034.

[10] Husakova J, Bem R, Fejfarova V, Jirkovska A, Woskova V, Jarosikova R, et al. Factors Influencing the Risk of Major Amputation in Patients with Diabetic Foot Ulcers Treated by Autologous Cell Therapy[J]. J Diabetes Res, 2022, 2022: 3954740.

[11] Arnadottir S D, Palsdottir G, Logason K, Arnardottir R H. [Lower Limb Amputations in Patients with Peripheral Arterial Disease and/or Diabetes in Iceland 2010-2019; revascularisation, comorbidities and risk factors][J]. Laeknabladid, 2024, 110(1): 20-27.

[12] Jude E B, Eleftheriadou I, Tentolouris N. Peripheral arterial disease in diabetes--a review[J]. Diabet Med, 2010, 27(1): 4-14.

[13] Biscetti F, Cecchini A L, Rando M M, Nardella E, Gasbarrini A, Massetti M, et al. Principal predictors of major adverse limb events in diabetic peripheral artery disease: A narrative review[J]. Atheroscler Plus, 2021, 46: 1-14.

[14] Xinyu Z, Mingjie G, Lili W, Chun D. Evaluation of Diabetic Lower Limb Arteriosclerosis Obliterative Disease and Risk Factors by Ultrasonography(In Chinese)[J]. Chinese J Ultrasound Med, 2016, 32(06): 544-546.

[15] Li L, Yuejuan C, Zhongjie P, Qinghong S, Yangchun L, Chengzhi W, et al. Research on the Treatment of Diabetic Lower Limb Arteriosclerosis Obliterans Disease(In Chinese)[M].

[16] Lei Z, Jianghao Y, Xinxi L, Ye T, Jun L. Risk factors and prediction model development for lower limb re-amputation in patients with type 2

diabetes-related lower extremity arterial disease(In Chinese)[J]. Chin J Vasc Surg（Electronic Version), 2025, 17(01): 55-60.

[17] Newhall K, Spangler E, Dzebisashvili N, Goodman D C, Goodney P. Amputation Rates for Patients with Diabetes and Peripheral Arterial Disease: The Effects of Race and Region[J]. Ann Vasc Surg, 2016, 30: 292-8.e1.

[18] Barnes J A, Eid M A, Creager M A, Goodney P P. Epidemiology and Risk of Amputation in Patients With Diabetes Mellitus and Peripheral Artery Disease[J]. Arterioscler Thromb Vasc Biol, 2020, 40(8): 1808-1817.

[19] Minc S D, Hendricks B, Misra R, Ren Y, Thibault D, Marone L, et al. Geographic variation in amputation rates among patients with diabetes and/or peripheral arterial disease in the rural state of West Virginia identifies areas for improved care[J]. J Vasc Surg, 2020, 71(5): 1708-1717.e5.

[20] Fereydooni A, Patel J, Dossabhoy S S, George E L, Arya S. Racial, ethnic, and socioeconomic inequities in amputation risk for patients with peripheral artery disease and diabetes[J]. Semin Vasc Surg, 2023, 36(1): 9-18.

[21] Fowler X P, Eid M A, Barnes J A, Gladders B, Austin A M, Goodney E J, et al. Trends of Concomitant Diabetes and Peripheral Artery Disease and Lower Extremity Amputation in US Medicare Patients, 2007 to 2019[J]. Circ Cardiovasc Qual Outcomes, 2023, 16(6): e009531.

[22] Min Y, Huisheng D. Risk Factors of Lower Extremity Atherosclerotic Occlusive Disease in Elderly Patients with Type 2 Diabetes Mellitus(In Chinese)[J]. Chinese General Practice, 2017, 20(01): 71-75.

[23] Olesen K K W, Gyldenkerne C, Thim T, Thomsen R W, Maeng M. Peripheral artery disease, lower limb revascularization, and amputation in diabetes patients with and without coronary artery disease: a cohort study from the Western Denmark Heart Registry[J]. BMJ Open Diabetes Res Care, 2021, 9(1).

[24] Lilja E, Gottsäter A, Miftaraj M, Ekelund J, Eliasson B, Svensson A M, et al. The impact of diabetes mellitus on major amputation among patients with chronic limb threatening ischemia undergoing elective endovascular therapy- a nationwide propensity score adjusted analysis[J]. J Diabetes Complications, 2021, 35(2): 107675.

[25] Ochoa Chaar C I, Malas M, Doros G, Schermerhorn M, Conte M S, Alameddine D, et al. The impact of diabetes mellitus on the outcomes of revascularization for chronic limb-threatening ischemia in the BEST-CLI trial[J]. J Vasc Surg, 2025, 81(2): 376-385.e3.

[26] Lejay A, Georg Y, Tartaglia E, Gaertner S, Geny B, Thaveau F, et al. Long-term outcomes of direct and indirect below-the-knee open revascularization based on the angiosome concept in diabetic patients with critical limb ischemia[J]. Ann Vasc Surg, 2014, 28(4): 983-9.

[27] Ishii H, Kumada Y, Takahashi H, Toriyama T, Aoyama T, Tanaka M, et al. Impact of diabetes and glycaemic control on peripheral artery disease in Japanese patients with end-stage renal disease: long-term follow-up study from the beginning of haemodialysis[J]. Diabetologia, 2012, 55(5): 1304-9.

[28] Jianping X, Xinhua X, Baoyu． F. Analysis of Influencing Factors of Type 2 Diabetes Complicated with Lower Extremity Arterial Occlusive Disease(In Chinese)[J]. J MedＲes, 2020, 49(03): 35-38.

[29] Higashi Y, Miyata T, Shigematsu H, Origasa H, Fujita M, Matsuo H, et al. Evaluation of Risk Factors for Major Amputation in Patients With Diabetes and Peripheral Artery Disease Receiving Antiplatelet Therapy　- Post Hoc Analysis of a Prospective Observational Multicenter Cohort Study (SEASON)[J]. Circ J, 2019, 83(9): 1929-1936.

[30] Liping Z, Jinxia L. Study on risk factors of type 2 diabetic mellitus complicated with

lower extremity arterial occlusive disease(In Chinese)[J]. Journal of Clinical Medicine Research and Practice, 2021, 6(23): 14-16.

[31] Mii S, Guntani A, Kawakubo E, Shimazoe H. Barthel Index and Outcome of Open Bypass for Critical Limb Ischemia[J]. Circ J, 2017, 82(1): 251-257.

[32] Tokuda T, Hirano K, Yamawaki M, Araki M, Kobayashi N, Sakamoto Y, et al. The Impact of Decline in Activities of Daily Living (ADL) of Patients With Critical Limb Ischemia (CLI) After Endovascular Treatment[J]. Vasc Endovascular Surg, 2018, 52(6): 411-417.

[33] Arya S, Binney Z O, Khakharia A, Long C A, Brewster L P, Wilson P W, et al. High hemoglobin A(1c) associated with increased adverse limb events in peripheral arterial disease patients undergoing revascularization[J]. J Vasc Surg, 2018, 67(1): 217-228.e1.

[34] Cha J J, Kim H, Ko Y G, Choi D, Lee J H, Yoon C H, et al. Influence of preprocedural glycemic control on clinical outcomes of endovascular therapy in diabetic patients with lower extremity artery disease: an analysis from a Korean multicenter retrospective registry cohort[J]. Cardiovasc Diabetol, 2020, 19(1): 97.

[35] Mcginigle K L, Kindell D G, Strassle P D, Crowner J R, Pascarella L, Farber M A, et al. Poor glycemic control is associated with significant increase in major limb amputation and adverse events in the 30-day postoperative period after infrainguinal bypass[J]. J Vasc Surg, 2020, 72(3): 987-994.

[36] Yunyun H, Xueying W. Relationship of Serum Bilirubin and HbA1c Levels with Lower Extremity Atherosclerotic Occlusive Disease in T2DM Patients(In Chinese)[J]. Jiangsu Med　2023, 49(09): 926-930.

[37] Liang K W, Kuo H N, Lee W L, Liu T J, Lin W W, Tsao C R, et al. Different Mid-Term Prognostic Predictors of Major Adverse Events in Diabetic and Nondiabetic Peripheral Artery Disease Presenting With Critical Limb Ischemia[J]. Angiology, 2016, 67(3): 287-91.

[38] Bosevski M, Krstevski G, Mitevska I, Antova E, Bosevska G. Hyperfibrinogenemia in Peripheral Arterial Disease: Coexistent and Independent Risk Factor (A Report of Two Cases and Review of Literature)[J]. Pril (Makedon Akad Nauk Umet Odd Med Nauki), 2018, 39(2-3): 93-96.

[39] Yuanyuan D, Qingfeng W, Lan L, Cong L, Jingxuan W, Junbo Z, et al. Association of preoperative plasma fibrinogen levels with adverse outcomes 1 year after endovascular revascularization in diabetes complicated with lower extremity arteriosclerosis obliterans(In Chinese)[J]. Journal of Xi'an Jiaotong University(Medical Sciences), 2025, 46(03): 463-471.

[40] Yang W, Jingmin O. Correlation between inflammatory markers and type 2 diabetes in patients with arteriosclerosis obliterans(In Chinese)[J]. Journal of Tongji University (Medical Science), 2023, 44(01): 57-61.

[41] Jingzhen L, Zheng Z, Liang L, Dan W, Yanwei S, Ning W. Homocysteine, Fibrinogen in Diabetic lower Limb Arteriosclerosis Occlusion of the Expression and Clinical Signifi cance(In Chinese)[J]. World Latest Medicne Information (Electronic Version) 2019, 19(A0): 15-17+21.

[42] Meloni M, Izzo V, Giurato L, Brocco E, Ferrannini M, Gandini R, et al. Procalcitonin Is a Prognostic Marker of Hospital Outcomes in Patients with Critical Limb Ischemia and Diabetic Foot Infection[J]. J Diabetes Res, 2019, 2019: 4312737.

[43] Govsyeyev N, Nehler M R, Low Wang C C, Kavanagh S, Hiatt W R, Long C, et al. Etiology and outcomes of amputation in patients with peripheral artery disease in the EUCLID trial[J]. J Vasc Surg, 2022, 75(2): 660-670.e3.

[44] Zhichao L, Yu M, Huiran D, En L. Correlation study between monocyte to high density lipoprotein ratio and arteriosclerosis obliterans of lower extremity in patients with type 2 diabetes mellitus(In Chinese)[J]. Chin J Arterioscler, 2020, 28(03): 242-246+252.

[45] Wenbin J, Guiming W, Xin D, Xing H. Relationship of serum CHI3L1 and HSP60 with restenosis following vascular intervention in T2DM patients with lower-extremity arteriosclerosis obliterans(In Chinese)[J]. Chin J Biomed Eng, 2022, 28(4): 442-445.

[46] Meili Z, Luli Y, Xueshi Y. Association of serum CysC, IMA and LP-PLA2 with these verity of lower extremity atherosclerotic occlusive disease in type

2 diabetes mellitus patients(In Chinese)[J]. Journal of Electrocardiology and Circulation, 2022, 41(01): 58-61.

[47] Qin Z, Chaoming P, Hong Y, Qi W. Clinical Value of Serum UA, HMGB1 and UAT in Predicting the Prognosis in Patients with Diabetes and Lower Extremity ASO(In Chinese)[J]. Int J Lab Med, 2021, 42(19): 2337-2341.

[48] Caixia A, Hui F, Yue Y, Xueling S. Correlation between serum high mobility group box 1 protein and in⁃sent restenosis in type 2 diabetic patients with lower limb arteriosclerosis obliterans(In Chinese)[J]. Chin J Diabetes, 2019, 27(06): 429-433.

[49] Bo S, Xu Z, Jiefeng Z, Wei W, Jicun Z, Yuehong Z. The relationship between VCAM-1 and restenosis after stent implantation in patients with type 2 diabetes mellitus and lower extremity arteriosclerosis obliterans(In Chinese)[J]. Chinese Medical Journal, 2020, 55(04): 406-409.

[50] Biscetti F, Nardella E, Rando M M, Cecchini A L, Bonadia N, Bruno P, et al. Sortilin levels correlate with major cardiovascular events of diabetic patients with peripheral artery disease following revascularization: a prospective study[J]. Cardiovasc Diabetol, 2020, 19(1): 147.

[51] Thott O, Granath F, Malmstedt J, Wahlgren C M. Editor's Choice - Dual Antiplatelet Therapy Improves Outcome in Diabetic Patients Undergoing Endovascular Femoropopliteal Stenting for Critical Limb Ischaemia[J]. Eur J Vasc Endovasc Surg, 2017, 53(3): 403-410.

[52] Shaofei S, Yufeng T, Linbao C, Bo Y. Combined anticoagulation and antiplatelet therapy for prophylaxis of restenosis after stent placement for leg atherosclerosis obliterans in diabetic patients(In Chinese)[J]. Chinese Journal of General Surgery, 2017, 25(03): 218-221.

[53] Bibo G. Application of cilostazol combined with bayaspirin in patients after diabetic lower extremity arteriosclerosis obliterans operation(In Chinese)[J]. Chinese Journal of Practical Medicine, 2019, 46(7): 97-100.

[54] Hsu C Y, Chen Y T, Su Y W, Chang C C, Huang P H, Lin S J. Statin Therapy Reduces Future Risk of Lower-Limb Amputation in Patients With Diabetes and Peripheral Artery Disease[J]. J Clin Endocrinol Metab, 2017, 102(7): 2373-2381.

[55] Lei Z. Study on Clinical Efficacy of Warfarin Combined with Cilostazol in the

Treatment and Prevention of Restenosis after Stent Implantation in Diabetic

Lower Extremity Arteriosclerosis Obliterans(In Chinese)[J]. Chinese Journal of Rational Drug Use, 2019, 16(11): 160-162+165.

[56] Rodionov R N, Peters F, Marschall U, L'hoest H, Jarzebska N, Behrendt C A. Initiation of SGLT2 Inhibitors and the Risk of Lower Extremity Minor and Major Amputation in Patients with Type 2 Diabetes and Peripheral Arterial Disease: A Health Claims Data Analysis[J]. Eur J Vasc Endovasc Surg, 2021, 62(6): 981-990.

[57] Lee H F, Chen S W, Liu J R, Li P R, Wu L S, Chang S H, et al. Major adverse cardiovascular and limb events in patients with diabetes and concomitant peripheral artery disease treated with sodium glucose cotransporter 2 inhibitor versus dipeptidyl peptidase-4 inhibitor[J]. Cardiovasc Diabetol, 2020, 19(1): 160.

[58] Hussain M A, Al-Omran M, Salata K, Sivaswamy A, Forbes T L, Sattar N, et al. Population-based secular trends in lower-extremity amputation for diabetes and peripheral artery disease[J]. Cmaj, 2019, 191(35): E955-e961.

[59] Griffin K E, Snyder K, Javid A H, Hackstadt A, Greevy R, Grijalva C G, et al. Use of SGLT2i Versus DPP-4i as an Add-on Therapy and the Risk of PAD-Related Surgical Events (Amputation, Stent Placement, or Vascular Surgery): A Cohort Study in Veterans With Diabetes[J]. Diabetes Care, 2025, 48(3): 361-370.

[60] Chang C C, Chen Y T, Hsu C Y, Su Y W, Chiu C C, Leu H B, et al. Dipeptidyl Peptidase-4 Inhibitors, Peripheral Arterial Disease, and Lower Extremity Amputation Risk in Diabetic Patients[J]. Am J Med, 2017, 130(3): 348-355.

[61] Caruso P, Angelino S, Matrone R, Scappaticcio L, Longo M, Volatile A, et al. Association of semaglutide with less limb events in people with type 2 diabetes and peripheral artery disease or foot ulcers: An observational study comparing matched cohorts[J]. Diabetes Obes Metab, 2025, 27(10): 5506-5513.

[62] Dubský M, Sojáková D, Husáková J, Němcová A, Fejfarová V, Jarošíková R, et al. Long-term efficacy of autologous cell therapy, repeated PTA and conservative treatment in people with diabetes and chronic limb-threatening ischaemia[J]. Diabetes Obes Metab, 2025, 27(9): 4729-4736.

[63] Wu J Y, Tu W L, Yu T, Liao K M, Lin Y M. Tirzepatide and major adverse limb events: Insights from a multicenter real-world analysis in PAD and diabetes patients[J]. Diabetes Res Clin Pract, 2025, 222: 112083.

[64] Freisinger E, Malyar N M, Reinecke H, Lawall H. Impact of diabetes on outcome in critical limb ischemia with tissue loss: a large-scaled routine data analysis[J]. Cardiovasc Diabetol, 2017, 16(1): 41.

[65] Aiello A, Anichini R, Brocco E, Caravaggi C, Chiavetta A, Cioni R, et al. Treatment of peripheral arterial disease in diabetes: a consensus of the Italian Societies of Diabetes (SID, AMD), Radiology (SIRM) and Vascular Endovascular Surgery (SICVE)[J]. Nutr Metab Cardiovasc Dis, 2014, 24(4): 355-69.

[66] Faglia E, Favales F, Quarantiello A, Calia P, Clelia P, Brambilla G, et al. Angiographic evaluation of peripheral arterial occlusive disease and its role as a prognostic determinant for major amputation in diabetic subjects with foot ulcers[J]. Diabetes Care, 1998, 21(4): 625-30.

[67] Lin J H, Jeon S Y, Romano P S, Humphries M D. Rates and timing of subsequent amputation after initial minor amputation[J]. J Vasc Surg, 2020, 72(1): 268-275.

[68] Shah S, Vanclay F, Cooper B. Improving the sensitivity of the Barthel Index for stroke rehabilitation[J]. J Clin Epidemiol, 1989, 42(8): 703-9.

[69] Quan H, Li B, Couris C M, Fushimi K, Graham P, Hider P, et al. Updating and validating the Charlson comorbidity index and score for risk adjustment in hospital discharge abstracts using data from 6 countries[J]. Am J Epidemiol, 2011, 173(6): 676-82.
